# CRISPR/Cas9‐Mediated Knockouts of the *ALG3* and *GNTI* in *N. benthamiana* and Their Application to Pharmaceutical Production

**DOI:** 10.1111/pbi.70326

**Published:** 2025-09-04

**Authors:** Dolgormaa Bataa, Hiroyuki Kajiura, Reimi Lai Sang Sawada‐Choi, Yukino Yamashita, Takeshi Ishimizu, Ryo Misaki, Atsushi Takeda, Kazuhito Fujiyama

**Affiliations:** ^1^ International Center for Biotechnology University of Osaka Osaka Japan; ^2^ Department of Biotechnology, Graduate School of Engineering University of Osaka Osaka Japan; ^3^ Department of Medical Technology and Clinical Engineering Gunma University of Health and Welfare Maebashi Japan; ^4^ Institute for Open and Transdisciplinary Research Initiatives (OTRI) University of Osaka Osaka Japan; ^5^ College of Life Sciences Ritsumeikan University Kusatsu Japan; ^6^ Osaka University Cooperative Research Station in Southeast Asia (OU:CRS), Faculty of Science Mahidol University Bangkok Thailand

**Keywords:** α1,3‐mannosyltransferase, CRISPR/Cas9, *N*‐acetylglucosaminyltransferase I, *N*‐glycoengineering, *Nicotiana benthamiana*

## Abstract

*N*‐Glycosylation critically influences the efficacy, safety and pharmacokinetic properties of biopharmaceuticals. Plant expression platforms offer multiple advantages for the production of *N*‐glycosylated proteins, but their use is impeded by the presence of plant‐specific *N*‐glycan epitopes, which raise concerns of possible immunogenicity to humans. In this study, *N*‐glycoengineered *Nicotiana benthamiana* plants that produce more homogeneous *N*‐glycans without plant‐specific epitopes were generated using multiplex CRISPR/Cas9 genome editing. To achieve this *N*‐glycosylation modification, ALG3 and GNTI, which function in *N*‐glycosylation processes in the ER and Golgi, respectively, were characterised, and single‐ and double‐knockout mutant plants were generated. Comprehensive *N*‐glycan profiling revealed that while the *ALG3*‐knockout plant line, *alg3*, maintained predominantly plant‐specific *N*‐glycans with altered mannose content, the *GNTI*‐knockout line, *gntI*, produced exclusively high‐mannose‐type *N*‐glycans. Notably, the *alg3gntI* double‐knockout mutants yielded highly uniform trimannosidic *N*‐glycans. To validate our *N*‐glycoengineering approach, we expressed two model biopharmaceuticals, Varlilumab (anti‐CD27 antibody) and β‐glucocerebrosidase (GCase), in wild‐type and mutant plants. While the antibodies expressed in *alg3* and *alg3gntI* showed a certain level of glucosylated endoplasmic reticulum‐type *N*‐glycan, with increased non‐*N*‐glycosylated heavy chains, GCase exhibited a more consistent *N‐*glycosylation profile, reflecting the engineered *N*‐glycosylation pathway. Our findings provide valuable insights into *N*‐glycan biosynthesis in *N. benthamiana* and demonstrate the potential of targeted *N*‐glycoengineering for producing biopharmaceuticals with more homogeneous mannose‐type *N*‐glycan profiles.

## 1 Introduction

*N‐*Glycosylation of biopharmaceuticals is critically important for their efficacy, safety and pharmacokinetic properties. The *N*‐glycan structures attached to therapeutic proteins significantly influence their stability, solubility, biological activity, immunogenicity and circulatory half‐life (Chen et al. 2022; Higel et al. 2019). For instance, proper *N*‐glycosylation of monoclonal antibodies is essential for effector functions such as antibody‐dependent cellular cytotoxicity and complement‐dependent cytotoxicity (Cambay et al. 2020). Regulatory authorities, such as the U.S. Food and Drug Administration (FDA) and European Medicines Agency, consider *N‐*glycosylation profiles as critical quality attributes, requiring comprehensive characterisation and batch‐to‐batch consistency (Luo and Zhang 2024; Szekrenyes et al. 2020; Taron and Duke 2015). Therefore, understanding and controlling *N*‐glycosylation is essential for developing safe and effective biopharmaceuticals with optimised therapeutic properties.

In this biopharmaceutical industrial era, plants are a promising biomanufacturing platform for recombinant proteins, offering several advantages over traditional expression systems. Plant‐based expression systems provide cost‐effective production, ease of scalability, absence of human pathogens, and the capacity to perform complex posttranslational modifications (Gecchele et al. 2015; Schillberg et al. 2019). Particularly, *Nicotiana benthamiana* produces considerably large amounts of biomass in a short period. In combination with the *Agrobacterium*‐mediated transient expression system, *N. benthamiana* has proven to be a valuable production host for various biopharmaceuticals, including antibodies, vaccines and therapeutic enzymes (Beritza et al. 2024; Tzfira and Citovsky 2006). Despite these advantages, the commercialisation of plant‐made pharmaceuticals has been hindered by several challenges, with plant‐specific *N*‐glycosylation patterns being the primary concern. The presence of plant‐specific β1,2‐xylose and α1,3‐fucose residues on *N*‐glycans can potentially induce immunogenic responses in humans and lead to rapid clearance of therapeutic proteins (Higel et al. 2019; Zhou and Qiu 2019) (Figure S1). These immunogenic concerns have motivated *N*‐glycoengineering efforts to humanise plant‐produced glycoproteins and improve their therapeutic efficacy and safety.

Earlier plant *N*‐glycoengineering studies focused on eliminating plant‐specific epitopes by downregulating the expression of β1,2‐xylosyltransferase (XYLT) and α1,3‐fucosyltransferase (FUCT) enzymes. In *N. benthamiana*, expression of *XYLT* and *FUCT* was downregulated using RNA interference (RNAi) technology, leading to undetectable levels of β1,2‐xylose and α1,3‐fucose residues on the *N*‐glycosylation site of a monoclonal antibody, 2G12 (Strasser et al. 2008). Building on this *N*‐glycoengineered platform, mammalian‐type *N*‐glycosylation was achieved through the expression of human glycosyltransferases. For instance, stable expression of human β1,4‐galactosyltransferase (GALT) resulted in β1,4‐galactosylated *N*‐glycans (Strasser et al. 2009). Moreover, mammalian‐type bisected and tri–/tetra‐antennary complex *N*‐glycans were obtained by either transient co‐expression or stable expression of human β1,4‐mannosyl‐β1,4‐*N*‐acetylglucosaminyltransferase (GnTIII), α1,3‐mannosyl‐β1,4‐*N*‐acetylglucosaminyltransferase (GnTIV) and α1,6‐mannosyl‐β1,6‐*N*‐acetylglucosaminyltransferase (GnTV) in *N. benthamiana* (Castilho et al. 2011; Nagels et al. 2011). Furthermore, α2,6‐sialylation was successfully achieved in *N. benthamiana* by co‐expressing six mammalian enzymes involved in the sialic acid biosynthetic and transfer pathway (Castilho et al. 2010). In parallel with the above line of research, clustered regularly interspaced short palindromic repeats (CRISPR) and CRISPR‐associated protein 9 (Cas9) were discovered and successfully applied to plants, enabling precise and efficient genome editing (Jinek et al. 2012; Li et al. 2013). Using the CRISPR/Cas9 tool, concurrent knockout of four genes of FUCT isoforms and two genes of XYLT isoforms was carried out in *N. benthamiana* (Jansing et al. 2019).

**FIGURE 1:**
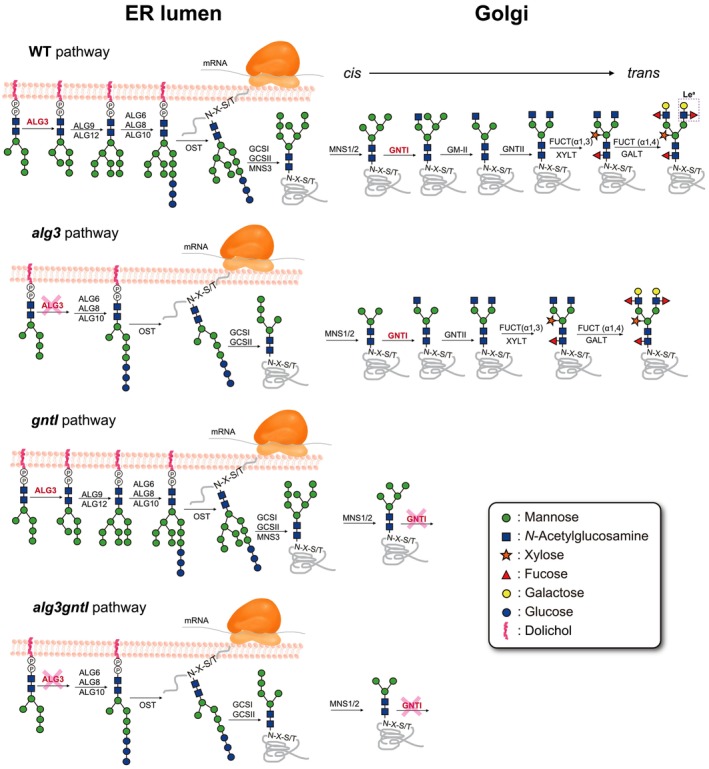
General *N*‐glycosylation pathway in the *N. benthamiana* WT and *alg3, gntI* and *alg3gntI*. The glycosylation pathway is illustrated starting from the intermediate M5^ER^ on the lumen side of the ER membrane. Dolichol phosphate (PP)‐linked oligosaccharide is subsequently synthetised by asparagine‐linked glycosylation (ALG) enzymes. The final dolichol PP‐linked oligosaccharide containing three glucose residues is transferred to the Asn (N) residues of the polypeptide chain by an oligosaccharyltransferase (OST) complex. Additional enzymes for *N*‐glycosylation maturation are as follows: α‐glucosidase I and II (GCSI and GCSII), ER α‐mannosidase I (MNS3), Golgi class I mannosidases (MNS1/2), *N*‐acytelglucosaminyltransferase I (GNTI), Golgi class II mannosidase (GMII), xylosyltransferase (XYLT), *N*‐acytelglucosaminyltransferase II (GNTII), α1,3 fucosyltransferase (FUCT (α1,3)), galactosyltransferase (GALT) and α1,4 fucosyltransferase (FUCT (α1,4)). Glycosyltransferases targeted in this study are written in pink. Specific symbols representing the sugar residues are displayed in the box.

Despite these extensive *N*‐glycoengineering efforts, mammalian *N*‐glycan structures are not always necessary or desired for all therapeutic proteins. This is particularly true for lysosomal enzyme replacement therapeutics, which require high‐mannose *N*‐glycans, as they are transported to lysosomes via the mannose‐6‐phosphate receptor pathway (Kornfeld 1987). To address this necessity, inhibitors or downregulators of the enzymes involved in the early stages of *N*‐glycan processing, such as ER α‐mannosidase I (MNS3) or *N*‐acetylglucosaminyltransferase I (GNTI), have been used to produce proteins with high‐mannose or pauci‐mannose *N*‐glycans that lack complex *N*‐glycan structures (Choi et al. 2018; Limkul et al. 2016). Moreover, T‐DNA insertion mutation of the α1,3‐mannosyltransferase (ALG3) in *A. thaliana* is reported to produce mostly paucimannosidic *N*‐glycans with reduced levels of plant‐specific *N*‐glycans (Henquet et al. 2008; Kajiura et al. 2010; Sariyatun et al. 2021). Furthermore, from a quality control perspective, not only structural *N*‐glycan modification but also the need to overcome *N*‐glycan heterogeneity remains a critical challenge. Notably, FDA‐approved placental‐derived β‐glucocerebrosidase (GCase) and recombinantly expressed GCase in carrot cell culture, Elelyso, maintain highly compact *N*‐glycan structures (Friedman et al. 1999; Friedman and Hayes 1996). Thus, modifications of *N*‐glycans to make them more homogeneous, or so that they contain only terminal mannose (Man) residues, have emerged as new challenges to be addressed. The ALG3 and GNTI enzymes are key targets for such *N*‐glycan engineering efforts, since they control important branching points in the endoplasmic reticulum (ER) and the Golgi in the *N*‐glycosylation pathway (Figure 1). The previous studies demonstrated that knockout of genes encoding these enzymes resulted in more uniform *N*‐glycans lacking plant‐specific epitopes without the need for extensive engineering of the *N*‐glycosylation pathway (Henquet et al. 2008; Herman et al. 2021; Veit et al. 2018). Therefore, the knockouts of *ALG3* and/or *GNTI* meet the criteria for the production of recombinant lysosomal enzymes with mannosylated and more homogeneous *N*‐glycans.

In this study, we focused on ALG3 and GNTI in *N. benthamiana* as two key *N*‐glycosylation enzymes. We identified the genes encoding these enzymes and characterised their activity through in vivo and in vitro assays using a heterologous expression system or recombinant proteins. We then generated single (*alg3* or *gntI*) and double (*alg3gntI*) knockout *N. benthamiana* plants using the multiplex CRISPR/Cas9 gene editing tool. *N*‐glycan profiles and the phenotypes of T_3_ generation mutants were characterised. To assess the impact of the engineered glycosylation pathway, two therapeutic proteins, Varlilumab (an anti‐CD27 antibody) and GCase, were transiently expressed in the wild‐type (WT) and mutant plants as model *N*‐glycoproteins, and the *N*‐glycosylation patterns were analysed. Our findings provided valuable insights into the roles of ALG3 and GNTI in *N*‐glycan biosynthesis and maturation in *N. benthamiana* and demonstrate their potential as targets for *N*‐glycoengineering to produce biopharmaceuticals with more uniform *N*‐glycosylation profiles.

## 2 Results

### 2.1 Identification of *NbALG3* and *NbGNTI* Genes and Their Functional Analysis

To generate *ALG3* and *GNTI* knockout mutants, we first identified the corresponding genes and their homologues in *N. benthamiana*. A database search using the Sol Genomics Network database (https://solgenomics.sgn.cornell.edu/) with ALG3 and GNTI of *Arabidopsis thaliana* as queries led to the identification of one candidate gene for *ALG3* and two homologues of *GNTI*: Niben101Scf01521g16013.1 for *ALG3*, which is hereinafter designated as *NbALG3*, and Niben101Scf04294g06042.1 and Niben101Scf01176g01032.1 for *GNTI*, which are hereinafter designated as *NbGNTI‐A* and *NbGNTI‐B*, respectively. *NbALG3* encodes 434 amino acids (49.2 kDa) and the product was predicted to be a transmembrane protein with 10 transmembrane regions by the TMHMM server (https://services.healthtech.dtu.dk/services/TMHMM‐2.0/). The amino acid sequence similarity between AtALG3 and NbALG3 was 65.4%, and NbALG3 had a C‐terminal KKXX sequence (Teasdale and Jackson 1996) as a putative ER retention signal (Figure S2), suggesting that NbALG3 functions as α1,3‐mannosyltransferase in the ER. *NbGNTI‐A* and *NbGNTI‐B* encode proteins of 421 (49.2 kDa) and 401 (46.5 kDa) amino acids, respectively. The amino acid sequence similarities between NbGNTI‐A and NbGNTI‐B were 91.8%, and 74.6% and 70.3% when compared to AtGNTI, respectively. Although no annotation regarding a transmembrane domain is currently available for the two NbGNTIs in the Sol Genomics Network database, sequence comparison with AtGNTI suggests that both NbGNTIs contribute to *N*‐glycosylation in the Golgi (Figure S3). Interestingly, focusing on the alignment of NbGNTIs, although most of the essential domains for catalytic activity, such as the UDP‐GlcNAc/Mn^2+^ binding site known as the DxD motif, were conserved, NbGNTI‐B lacked one substrate binding site (Figure S3) (Gordon et al. 2006), implying that NbGNTI‐B might have no GNTI activity.

**FIGURE 2:**
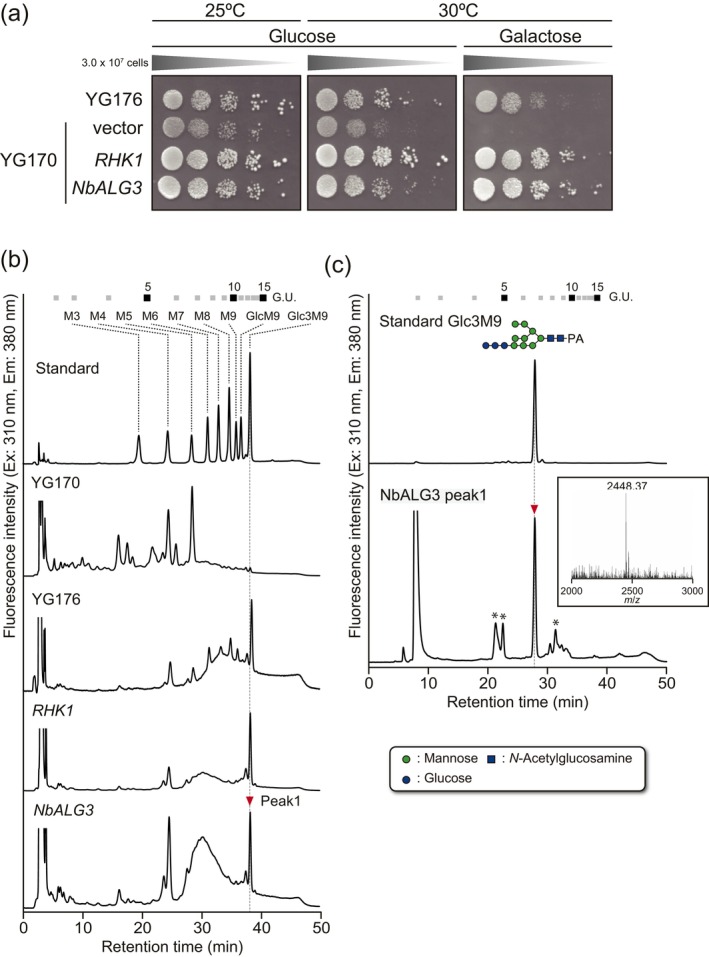
Functional analysis of NbALG3. (a) Complementation analysis of *S. cerevisiae* YG170 strains by NbALG3. Transformants harbouring each expression vector inserted with the cDNA driven under the control of the *GAL1* promoter were spotted in 10‐fold dilutions on plates containing glucose or galactose and incubated at 30°C for 2 days. YG176, a single *stt3* deletion strain, was able to grow, whereas YG170, which had a deletion of both *alg3* and one of the subunits of oligosaccharyltransferase, *stt3*, could not grow at 30°C. *RHK1*: ALG3 gene from the RHK1 (Resistance Hancenula Killer 1) mutant strain of *S. cerevisiae* was used as a control. (b) Analysis of the lipid‐linked oligosaccharide biosynthesised in each transformant. PA‐labelled oligosaccharides, after acid hydrolysis and purification of the lipid‐linked oligosaccharides from the indicated yeast strains, were separated by size‐fractionation HPLC. Numbers at the top represent the elution positions of glucose units on the basis of the elution times of PA‐isomalto‐oligosaccharides with degrees of polymerisation from 3 to 15. M3‐GlcM9 as shown in the chromatogram in Standard indicate the Man_x_GlcNAc_2_‐PA and Glc_x_Man_9_GlcNAc_2_‐PA. The symbols used for *N*‐glycan structures are shown in a small window. (c) Determination of the peak indicated by red inverted triangle in (b). The peak was collected and further analysed by reverse phase (RP)‐HPLC and LC–MS/MS analysis. Small window indicates the molecular mass of the major peak detected in LC–MS/MS analysis. The asterisks indicate peaks that do not correspond to PA‐glycans.

To examine each activity, heterologous expression systems were used. First, each cDNA was cloned from 5‐week‐old *N. benthamiana* leaves and inserted into expression vectors. For NbALG3, the activity was confirmed by the complementation test using a *Saccharomyces cerevisiae* *Δalg3* temperature‐sensitive mutant strain, YG170 (Kajiura et al. 2010; Zufferey et al. 1995), under the control of the galactose‐inducible (*GAL1*) promoter. NbALG3 rescued the temperature sensitivity of YG170, in the same manner as *S. cerevisiae* ALG3 (RHK1), in the presence of galactose, whereas YG170 and YG170 carrying the control expression vector could not grow in the presence of glucose (Glc) under the normal growth temperature (Figure 2a). It should be noted that YG170 expressing RHK1 exhibited a leaky phenotype, as even low levels of RHK1 expression were sufficient to complement the ALG3 deficiency in YG170 due to the strong activity of NbALG3 induced by trace amounts of intracellular galactose (Kajiura et al. 2010). These results suggested that NbALG3 has the same function as RHK1. To further characterise the function of NbALG3, the biosynthesis of core oligosaccharides on dolichol in the ER was confirmed (Figure 2b,c). YG170 accumulates Man_5_GlcNAc_2_^ER^ (M5^ER^), an intermediate of the lipid‐linked oligosaccharide, while YG170 expressing *RHK1* or *NbALG3* exhibited an accumulation of the final product of the full‐length oligosaccharide in the ER, Glc_3_Man_9_GlcNAc_2_ (Glc3M9) (GlcNAc, *N*‐acetylglucosamine) (Figure 2b,c and Figure S1). These results indicated that NbALG3 is *N. benthamiana* α1,3‐mannosyltransferase.

**FIGURE 3:**
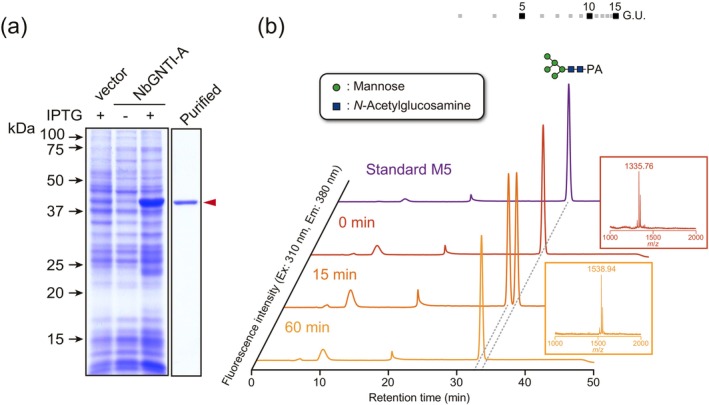
Expression and activity assay of recombinant NbGNTI‐A. (a) CBB staining of recombinant NbGNTI‐A. The crude cell lysate of *E. coli* expressing NbGNTI‐A and purified NbGNTI‐A using a Co^2+^ resin column were separated by SDS‐PAGE and visualised. The red triangle indicates NbGNTI‐A. (b) The HPLC profile of the recombinant NbGNTI‐A reaction product. The reaction products of NbGNTI‐A using M5‐PA and UDP‐GlcNAc as the acceptor and donor substrates, respectively, were analysed by RP‐HPLC and compared with the authentic PA‐sugar chain, GNM5‐PA. Small windows indicate the molecular mass of the major peak detected in each analysis. The peaks represent [M + Na]^+^ ions derived from the ion, *m/z* 1335.76 and 1538.94, which correspond to Hex_5_HexNAc_2_‐PA and Hex_5_HexNAc_3_‐PA, respectively. Numbers at the top represent the elution positions of glucose units.

To characterise NbGNTIs, heterologous recombinant proteins produced in *Escherichia coli* were used. Transmembrane domain‐truncated NbGNTI‐A was successfully produced and purified using a Co^2+^ column (Figure 3a). The purified protein was functionally active and exhibited GlcNAc transfer activity (Figure 3b), indicating that NbGNTI‐A is *N*‐acetylglucosaminyltransferase I in *N. benthamiana*. On the other hand, NbGNTI‐B was also produced as a soluble protein but could not be purified. Therefore, we tested its enzymatic activity in the crude extract. As hypothesised, the crude sample containing NbGNTI‐B did not exhibit any GNTI activity, probably due to the absence of an essential domain required for its function (Figures S3 and S4). These results further confirmed that *N. benthamiana*, despite its allotetraploid genome, possesses a single gene locus encoding each of the *ALG3* and *GNTI*.

### 2.2 CRISPR/Cas9‐Mediated Knockout of *N. benthamiana ALG3* and *GNTI*

**FIGURE 4:**
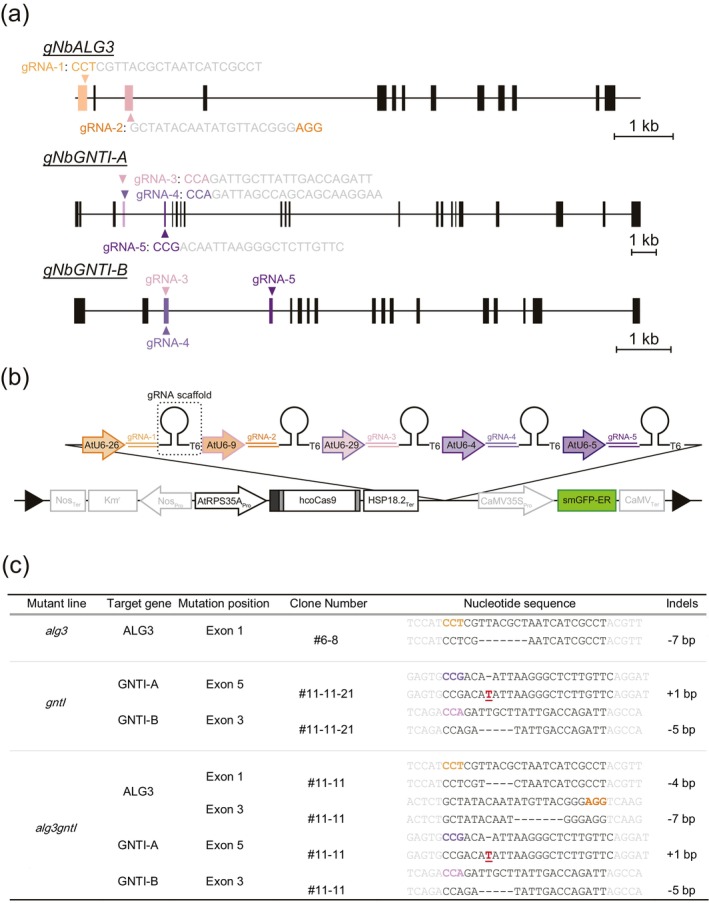
Generation of the *N. benthamiana alg3*, *gntI* and *alg3gntI*. (a) Schematic representation of CRISPR/Cas9 target sites in selected genes. The gRNAs targeting exons are shown in colours, untargeted exons are shown as black boxes, and introns are shown as black lines. gRNAs are designed for each gene and the PAM sequences in the target sites are highlighted in colours. (b) The multiplex CRISPR/Cas9 plant expression cassette. The human codon‐optimised Cas9 with N‐terminal 3 × FLAG and SV40 nuclear localisation signal (NLS) and C‐terminal nucleoplasmin NLS was constructed under the control of the *A. thaliana* Ribosomal protein S5A (AtRPS5A) promoter. A total of five spacer sequences (illustrated as coloured parallel lines) targeting the ALG3 and GNTI genes were inserted into pBluescript (pBS)‐U6‐based plasmids harbouring the single‐guide RNA scaffold (illustrated as a black loop). The U6‐sgRNA cassettes were then inserted into the multiplex CRISPR/Cas9 expression cassette at the *Bsa*I restriction enzyme sites by Golden Gate Cloning. NOS_Ter_: Nopaline synthase gene terminator, Kan^r^: Kanamycin resistance gene, NOS_Pro_: Nopaline synthase gene promoter, HSP18.2_Ter_: Heat shock protein 18.2 terminator from *A. thaliana* , LB: T‐DNA left borfer, RB: T‐DNA right border. (c) Genotype of the T3 generation *N. benthamiana alg3*, *gntI* and *alg3gntI* plants. The occurring homologous mutations are shown in the indels column. Insertions are highlighted in red and deletions are shown as hyphens.

To successfully engineer *N*‐glycan structures in *N. benthamiana* using CRISPR/Cas9, we first verified the genome sequences of *NbALG3* and *NbGNTI*s because the genome sequence of the *N. benthamiana* strain used often differs from the sequence in the database. The sequencing results revealed the nucleotide differences in *NbALG3*. With these differences in hand, we then carefully selected two gRNA target sites for *NbALG3* and three for *NbGNTIs* after comprehensive screening based on their genomic positions (Figure 4a) and minimal off‐target potential, as predicted by the CRISPRdirect tool (Naito et al. 2015). It should be noted that although *NbGNTI‐B* encoding protein had no in vitro GNTI activity, it might have in vivo activity. To eliminate this possibility, the gRNA sequence for *NbGNTI‐A* was designed to also be effective for *NbGNTI‐B*. A total of five gRNAs targeting the *NbALG3* and *NbGNTI* genes were incorporated into a single multiplex CRISPR/Cas9 expression vector by Golden Gate Cloning (Figure 4b), and then the vector was introduced into *Agrobacterium tumefaciens* GV3101 carrying the pSoup vector and used for stable transformation of WT *N. benthamiana*.

A three‐step screening method was employed to identify single (*alg3* or *gntI*) and double (*alg3gntI*) knockout mutants across all T_0_, T_1_, T_2_ and backcrossed plants. Previous studies strongly suggested that the loss of GNTI activity eliminated the plant‐specific β1,2‐xylose and α1,3‐fucose epitopes (Herman et al. 2021; Limkul et al. 2016; Strasser et al. 2005). Therefore, using the immunoreactivity of anti‐horseradish peroxidase antibody (anti‐HRP) to the plant‐specific *N*‐glycan epitopes (Wuhrer et al. 2005), GNTI activity in the mutant plants was assessed by Western blotting. Then the *NbALG3* target amplicon of selected plants was sequenced. Lastly, the sequences of the *NbGNTI‐A* and ‐*B* genes were checked by Sanger sequencing. Through this screening method, 17 T_0_ candidate mutant plants were identified and 5 putative mutant plants were selected. Subsequently, a total of 120 T_1_ plants were grown from the selected plants and screened thoroughly. As a result, mutant strain 6–8 was identified as a T_1_ *alg3* plant and strain 11–11 was identified as a T_1_ *alg3gntI* plant. Seeds were harvested from those mutants, and then the T_2_ and T_3_ plants were grown and the mutations of the targeted genes were confirmed once again. The T_3_ *N. benthamiana alg3* carried a 7‐nucleotide deletion located at +3 from the NGG protospacer adjacent motif (PAM) in exon 1. In the T_3_ *N. benthamiana alg3gntI*, the *NbALG3* gene carried a 4‐nucleotide deletion in exon 1 and a 7‐nucleotide deletion in exon 3, both occurring at position +4 from the PAM. Additionally, this mutant carried mutations in both *NbGNTI* genes: a 1‐nucleotide insertion at position +4 from the PAM in exon 5 of *NbGNTI‐A* and a 5‐nucleotide deletion at position +3 from the PAM in exon 3 of *NbGNTI‐B*. All induced mutations were frameshift mutations. The *gntI* plant could not be obtained from the T_0_ plants, presumably due to the strong effect of the gRNAs for *NbALG3*; therefore, this plant was generated by cross‐pollination between T_2_ *alg3gntI* and WT. In all, 225 F_1_ plants were screened, and strain 11‐11‐21 was identified as *N. benthamiana gntI* carrying the same frameshift mutation of both *NbGNTI* genes of the *alg3gntI*, and intact *NbALG3* gene was obtained (Figure 4c). Overall, the multiplex CRISPR/Cas9 genome editing successfully generated the single and double mutants carrying the homologous frameshift mutations leading to the disruption of the correct protein synthesis.

### 2.3 Whole *N*‐Glycan Profiles of the *N. benthamiana* WT, alg3, gntI and alg3gntI

**FIGURE 5:**
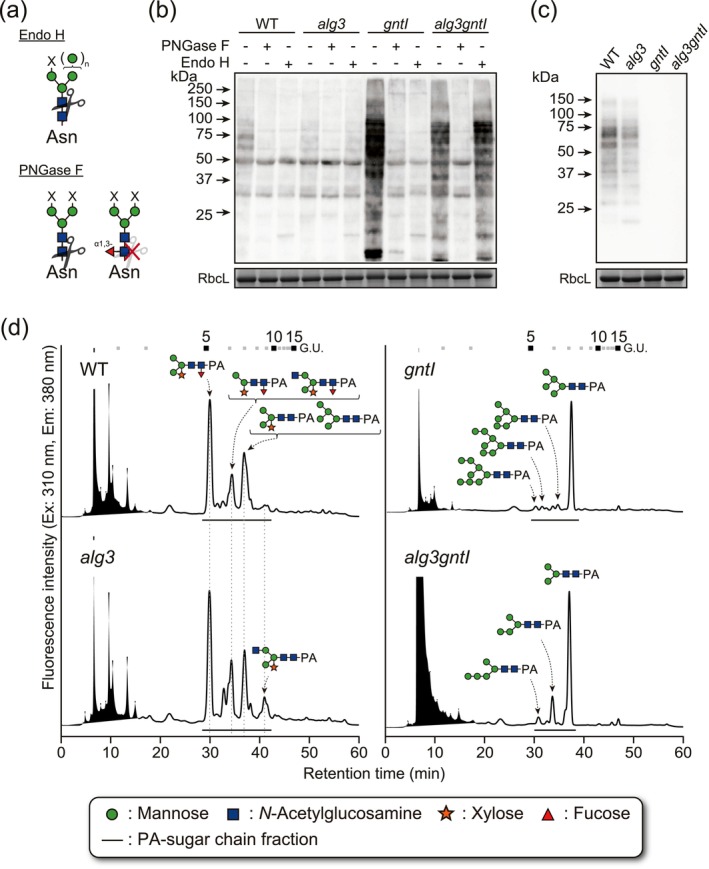
Analysis of the *N*‐glycan profiles in the *N. benthamiana* WT, T_3_ generation *alg3*, *gntI* and *alg3gntI*. (a) Schematic representations of the substrate specificities of Endo H and PNGase F; X can be single mannose/ *N*‐acetylglucosamine residue or elongated *N*‐glycan arm. (b) Total soluble protein extracts were digested with PNGase F and Endo H and separated by SDS‐PAGE. The results for the ConA‐probed lectin blot and the CBB‐stained large subunit of RuBisCO (RbcL) used as a loading control are shown. (c) Total soluble protein extracts were separated by SDS‐PAGE and the presence of plant‐specific‐type *N*‐glycans is visualised by anti‐HRP western blot. (d) Total *N*‐glycan profiles in the WT and T_3_ *N. benthamiana* mutants. Total *N*‐glycans were extracted, hydrolyzed, PA‐labelled and analysed using RP‐HPLC. Glucose oligomer standards are shown at the top of the chromatogram. The underlined peaks were collected and subjected to LC–MS/MS analysis. The main *N*‐glycans identified from the peaks are shown. Identified *N*‐glycan structures from each peak are displayed.

First, to investigate the *N‐*glycosylation profiles of the WT, *alg3*, *gntI* and *alg3gntI* plants, total soluble protein extracts were digested with the peptide‐*N*‐glycosidase F (PNGase F) and endoglycosidase H (Endo H) and visualised by blotting with concanavalin A (ConA) lectin, which has an affinity for the non‐reducing α‐mannose residues (Faye and Chrispeels 1985). PNGase F is unable to cleave complex *N*‐glycans from the *N*‐glycoprotein containing α1,3‐fucose residues, while Endo H specifically cleaves high‐mannose‐type *N*‐glycans from polypeptides, with ManGlcNAc_2_, Man_2_GlcNAc_2_ and Man_3_GlcNAc_2_ (M3) structures being exceptions to its enzymatic activity (Figure 5a) (Tretter et al. 1991; Trimble and Tarentino 1991). The WT and *alg3* plants showed similarly lower binding with the ConA lectin regardless of the glycosidase digestions, suggesting that both the WT and *alg3* plants contained mainly complex plant‐specific *N*‐glycans and only a small amount of mannosidic *N‐*glycans (Figure 5b). On the other hand, protein samples of the non‐digested *gntI* and *alg3gntI* mutants showed high affinity to the ConA, indicating an abundance of the mannosidic structure. This affinity of the *gntI* sample was diminished after the digestion with PNGase F and Endo H, whereas the affinity of the *alg3gntI* sample to the ConA was still present after Endo H digestion. In parallel, plant‐specific *N*‐glycan epitopes of the WT, *alg3*, *gntI* and *alg3gntI* plants were checked by anti‐HRP western blot. Only the WT and *alg3* total proteins bound with the anti‐HRP antibody (Figure 5c). These results demonstrated that the plant‐specific β1,2‐xylose and α1,3‐fucose epitopes were completely absent in the *gntI* and *alg3gntI* plants.

To provide more detailed *N‐*glycosylation profiles for the WT, *alg3*, *gntI* and *alg3gntI* plants, total *N‐*glycans were released from *N*‐glycoproteins by hydrazinolysis and labelled with 2‐aminopyridine (PA). The PA‐labelled *N*‐glycans were further separated by RP‐HPLC with a C18 column (Figure 5d); the PA *N‐*glycan peaks were collected, and the structures were further elucidated by the LC–MS/MS. In accordance with the ConA lectin blotting results, the WT and *alg3* mutant plants showed similar *N‐*glycan profiles, with 20.0% and 16.0% mannosidic *N‐*glycans and 80.0% and 84.0% plant‐specific *N‐*glycans, respectively. However, there was a difference in the composition of the *N‐*glycans of *alg3* and WT plants. Due to loss of the α1,3 mannosyltransferase activity in the *alg3* mutant, ER‐derived *N‐*glycan types—i.e., M5^ER^‐PA, GlcM5^ER^‐PA and M4^ER^‐PA—were detected at levels of 1.4%, 1.6% and 5.4%, respectively (Table 1 and Figure S1). As expected, disruption of GNTI synthesis resulted in a uniformly high‐mannose profile consisting of 100% mannosidic *N*‐glycans, with 80.1% of these being M5‐PA. Lastly, disruption of both ALG3 and GNTI resulted in a uniquely uniform *N‐*glycan composition of 86.2% M3‐PA, 12.6% M4^ER^‐PA and 1.3% M5^ER^‐PA. These results indicated that loss of both the ALG3 and GNTI enzymes completely arrested the *N‐*glycan maturation in the ER and Golgi, resulting in accumulation of highly uniform trimannosyl *N*‐glycans in *N. benthamiana*.

**TABLE 1:** Relative amounts of *N‐*glycan structures detected in the *N. benthamiana* WT and T_3_ generation *alg3*, *gntI* and *alg3gntI.*

Structure		Ratio (%)			
		WT	*alg3*	*gntI*	*alg3gntI*
Mannosidic type	M3	—	6.9	—	86.2
	M4	—	5.4	6.1	12.6
	M5	6.1	1.4	80.1	1.3
	M6	3.9	—	3.9	—
	M7A	2.3	—	2.4	—
	M7B	1.8	—	2.3	—
	M8A	2.9	—	3.5	—
	M8B	0.6	—	—	—
	M9	2.5	—	1.7	—
Glucosylated type	GlcM5	—	1.6	—	—
	Glc2M5	—	0.4	—	—
	Glc3M5	—	0.4	—	—
**Plant‐specific type**					
	M3X	11.2	4.3	—	—
	^M^MFX	6.8	3.6	—	—
	M3FX	58.4	54.5	—	—
Terminal GlcNAc residue(s)	_GN_M3X	—	1.6	—	—
	^GN^M3X	—	0.8	—	—
	_GN_M3FX	—	3.2	—	—
	^GN^M3FX	3.7	13.3	—	—
	GN2M3FX	—	1.9	—	—
Galactosylated type	GalGN2M3FX	—	0.1	—	—
	GalFGN2M3FX	—	0.7	—	—
**Total**					
Mannosidic type		20.0	16.0	100	100
Plant‐specific type		80.0	84.0	—	—

### 2.4 Phenotypic Analysis of Single and Double Mutants

**FIGURE 6:**
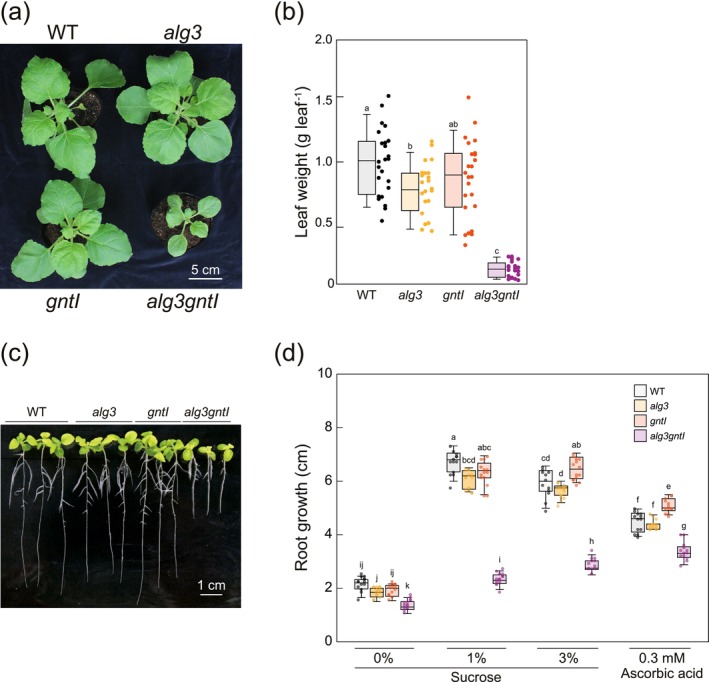
Phenotypic analysis of *N. benthamiana alg3*, *gntI* and *alg3gntI*. (a) Top angle image of the 5‐week‐old WT and T_3_ *N. benthamiana* mutants. (b) Boxplot of fresh leaf weight of the WT and mutant plants; median and individual values were plotted. (c) Root growth comparison of the WT and mutant seedlings. Seven‐day‐old seedlings on MS plates were transferred to rectangle MS plates with 3% sucrose and grown an additional 10 days. (d) Boxplot of the root length of the WT and mutant seedlings grown and transferred to an MS plate with 0%, 1% or 3% sucrose and 0.3 mM ascorbic acid supplement; median and individual values are plotted. The letters above each plot represent the significance of the difference between any two groups. A one‐way ANOVA was performed to compare the means between groups. The results showed a significant difference among the groups (*p* < 0.05). Tukey's HSD post hoc test was conducted for pairwise comparisons.

*N*‐Glycosylation deficiency might induce some growth defect(s). To address this possibility, the growth of mutants was assessed. The growth of 5‐week‐old T_3_ mutant plants was evaluated by measuring leaf weight. While the *alg3* and *gntI* plants showed no difference in leaf weight compared to the WT, *alg3gntI* exhibited significantly smaller leaves. In accordance with the leaf size, the *alg3* and *gntI* mutants displayed healthy growth (Figure 6a,b). In contrast, the double disruption of *GNTI* and *ALG3* caused severe growth impairments, including curled leaves and defective root development (Figure 6c). Although there was no obvious difference in the shoot‐growth phenotype between the *A. thaliana* WT and the mutants, root growth impairment was observed in the *cglalg3* mutant (*cgl* is a *GNTI* mutant of *A. thaliana* ) (Figure S5a,b). To further investigate these root development defects in *N. benthamiana*, mutant plants were examined under four different conditions: MS medium with 0%, 1% or 3% sucrose, and MS medium supplemented with 0.3 mM ascorbic acid to modulate the possible ER stress‐derived oxidative stress. The root growth responses of the WT, *alg3* and *gntI* plants were not substantially different from each other across all conditions. However, the *alg3gntI* exhibited impaired root growth in MS medium containing adequate sucrose (1% and 3%). Notably, the addition of ascorbic acid partially rescued this defective root phenotype in *alg3gntI* (Figure 6d). However, in the case of *A. thaliana* , the ascorbic acid addition had no effect on the defective root phenotype in the *cglalg3* mutant (Figure S5c,d). These results show how certain changes in *N*‐glycosylation led to growth defects in certain species, highlighting the intricate relationship between plant *N*‐glycosylation machinery and physiology.

### 2.5 Investigating the *N*‐Glycosylation Profiles of the Monoclonal Antibody Varlilumab

**FIGURE 7:**
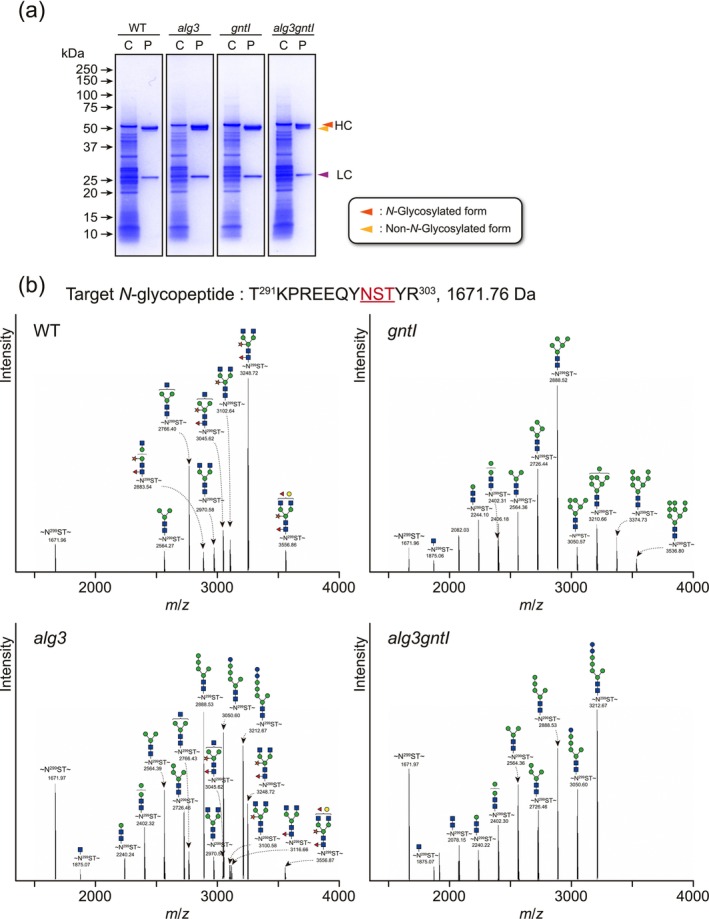
Analysis of *N*‐glycosylation of the model protein, Varlilumab. (a) Transiently expressed Varlilumab was purified, followed by separation by SDS‐PAGE, and visualised by CBB. C and P stand for crude and purified samples, respectively. Triangles indicate the heavy chain (HC) and light chain (LC) of Varlilumab. (b) *N*‐Glycosylation analysis of Asn299 in Varlilumab. The band corresponding to the heavy chain of Varlilumab was excised from the CBB‐stained gel, followed by in‐gel trypsin digestion and nanoLC‐MS/MS analysis. All signals of *m/z* corresponding to *N*‐glycopeptide and the *N*‐glycan structures are shown. The symbols used for *N*‐glycan structures are the same as in Figure 5d. The ratios of *N*‐glycans are shown in Table 2. The molecular weight of the peptide is indicated next to its amino acid sequence.

Many therapeutic proteins have already been produced in plants. Among them, the immunoglobulin G (IgG)‐based monoclonal antibodies are good candidates for evaluating changes in *N*‐glycosylation, as most IgGs have biantennary *N*‐glycans with β1,2‐xylose and/or α1,3‐fucose residues, such as GN2M3FX (Figure S1) (Chen et al. 2022). To examine the *N*‐glycosylation changes in the WT and mutant strains used in this study, we chose the IgG‐based monoclonal antibody Varlilumab. Varlilumab (CDX‐1127) is a fully human monoclonal antibody that targets CD27, a receptor involved in T‐cell activation, and has been investigated as an immunotherapeutic agent and transiently produced in *N. benthamiana* (Burris et al. 2017; Nguyen et al. 2023). The antibody contains a single *N*‐glycosylation site in the Fc region of the heavy chain, typically at N299 (^291^TKPREEQYNSTYR^303^, where the underlined Asn represent the *N*‐glycosylation site), which makes it suitable for examining *N*‐glycosylation changes. Varlilumab was transiently expressed and purified from the WT and mutant *N. benthamiana* leaves using a MonoSpin ProA spin column (Figure 7a). The purification yield of Varlilumab per mg of total soluble protein was highest in the *alg3gntI* mutant (17.4 μg/mg), followed by the *alg3* mutant (10.3 μg/mg), while the *WT* and *gntI* plants showed comparable but lower yields (6.0 and 6.1 μg/mg, respectively) (Table S1). Trypsin‐digested products of purified Varlilumab, followed by nanoLC–MS/MS analysis, demonstrated that the Fc region of the WT plant‐expressed antibody contained 62.8% plant‐specific *N‐*glycans, with the complex biantennary structure Hex_3_Pent_1_DeoxyHex_1_HexNAc_4_ which was most likely GN2M3FX, representing the predominant *N*‐glycoform at 39.6% of the total profile (Table 2). In contrast, the Fc region of Varlilumab produced in the *alg3* mutant predominantly possessed the ER‐derived immature *N‐*glycans, with a distribution comprising 7.0% M4^ER^, 17.2% M5^ER^, 15.3% GlcM5^ER^ and 13.7% Glc2M5^ER^ structures. Only 13.6% of *N*‐glycosylated peptide contained plant‐specific *N‐*glycans. In the ER, *N*‐glycosylated protein folding is facilitated by the chaperones calnexin and calreticulin, which bind specifically to GlcM9^ER^ *N*‐glycans (Lederkremer 2009). Another protein, malectin (an ER quality control‐associated lectin), is known to interact with Glc2M9^ER^‐glycosylated proteins (Feng et al. 2022). The absence of Glc3M5^ER^ *N*‐glycan in *alg3* plant can be attributed to the crucial role of trimming the first α1,3‐glucose residue in the proper folding of Varlilumab. In the case of Varlilumab produced in *gntI* plants, all the *N‐*glycans were mannosidic, with the M5 structure predominating at 32.2% of total *N*‐glycans. On the other hand, Varlilumab produced in *alg3gntI* plants had the ER‐derived immature *N‐*glycan profile similar to that of *alg3*, but without any plant‐specific‐type *N*‐glycans. The predominant *N‐*glycans of the *alg3gntI* were 12.1% M3, 16.8% M5^ER^, 11.8% GlcM5^ER^ and 21.3% Glc2M5^ER^ structures. Notably, CBB staining of the purified Varlilumab and the nanoLC–MS/MS result showed the non‐*N*‐glycosylated heavy chain amount was the highest, 13.9% in *alg3gntI* followed by 9.9% in *alg3*, 5.6% in WT and 4.0% in *gntI* (Figure 7a and Table 2). The *N‐*glycosylation profile of Varlilumab confirmed that disruption of the GNTI synthesis intercepted the action of plant‐specific glycosyltransferases. On the other hand, disruption of the ALG3 synthesis led to a considerably high level of immature ER‐derived *N*‐glycosylation of Varlilumab.

**TABLE 2:** *N*‐Glycosylation compositions on the heavy chain *N*‐glycopeptide (N299) of Varlilumab.

Structure			Ratio (%)			
	Composition	Abbreviation	WT	*alg3*	*gntI*	*alg3gntI*
	Peptide		5.6	9.9	4.0	13.9
	HexNAc_1_	GlcNAc	—	1.0	1.9	1.8
	HexNAc_2_	GlcNAc2	—	—	—	2.5
Mannosidic type	Hex_1_HexNAc_2_	M1	—	2.2	8.7	3.8
	Hex_2_HexNAc_2_	M2	—	5.5	5.4	7.2
	Hex_3_HexNAc_2_	M3	4.1	9.3	10.2	12.1
	Hex_4_HexNAc_2_	M4	—	—	17.5	—
	Hex_5_HexNAc_2_	M5	—	—	32.2	—
	Hex_6_HexNAc_2_	M6	—	—	4.2	—
	Hex_7_HexNAc_2_	M7A, B	—	—	7.9	—
	Hex_8_HexNAc_2_	M8A, B	—	—	5.9	—
	Hex_9_HexNAc_2_	M9	—	—	2.2	—
**ER‐derived type**						
	Hex_4_HexNAc_2_	M4^ER^	—	7.0	—	8.8
	Hex_5_HexNAc_2_	M5^ER^	—	17.2	—	16.8
Terminal Glc type	Hex_6_HexNAc_2_	GlcM5^ER^	—	15.3	—	11.8
	Hex_7_HexNAc_2_	Glc2M5^ER^	—	13.7	—	21.3
Terminal GlcNAc type	Hex_3_HexNAc_3_	GNM3	22.4	2.9	—	—
	Hex_3_HexNAc_4_	GN2M3	5.0	2.4	—	—
**Plant‐specific type**						
Terminal GlcNAc type	Hex_3_Pent_1_HexNAc_4_	GN2M3X	6.6	1.5	—	—
	Hex_3_DeoxyHex_1_HexNAc_4_	GN2M3F	—	1.4	—	—
	Hex_2_DeoxyHex_1_Pent_1_HexNAc_3_	GNM2FX	3.9	—	—	—
	Hex_3_DeoxyHex_1_Pent_1_HexNAc_3_	GNM3FX	8.3	1.8	—	—
	Hex_3_DeoxyHex_1_Pent_1_HexNAc_4_	GN2M3FX	39.6	7.7	—	—
Galactosylated type	Hex_4_DeoxyHex_2_Pent_1_HexNAc_4_	GalFGN2M3FX	4.3	1.2	—	—
**Total**						
Mannosidic type			4.1	70.2	94.1	81.8
ER‐derived type			—	53.2	—	58.7
Plant‐specific type			62.8	13.6	—	—

### 2.6 Production of Lysosomal Storage Disease Therapeutics With Precisely Engineered, Highly Mannosylated *N*‐Glycans

**FIGURE 8:**
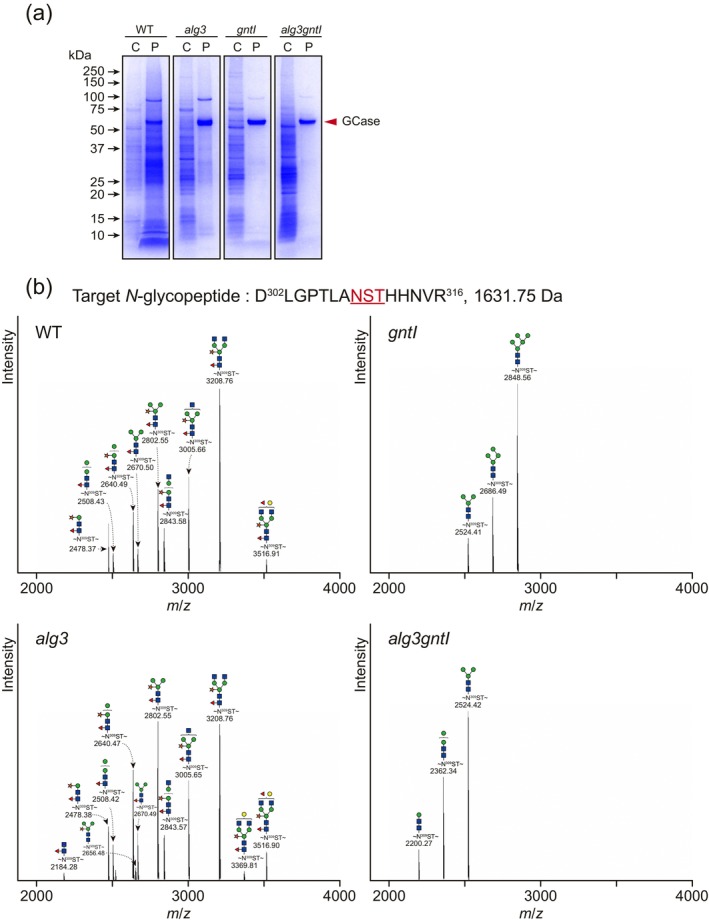
*N*‐Glycosylation analysis of GCase. (a) Transiently expressed GCase was purified, followed by SDS‐PAGE separation and CBB staining. C and P stand for crude and purified samples, respectively. (b) *N*‐Glycosylation analysis of Asn309 in GCase. The target band was excised from the CBB‐stained gel and trypsin‐digested. Tryptic peptides were analysed by nanoLC‐MS/MS. All *m/z* signals and corresponding *N*‐glycan structures of *N*‐glycopeptides are displayed. The symbols used for *N*‐glycan structures are shown in a small window. The ratios of *N*‐glycans and other *N*‐glycosylation sites are shown in Table 2 and Figure S6, respectively. The molecular weight of the peptide is indicated next to its amino acid sequence.

GCase is a lysosomal enzyme that catalyses the hydrolysis of glucosylceramide to glucose and ceramide. Mutations in the encoding gene cause Gaucher's disease (Beutler 1988). The enzyme contains five potential *N‐*glycosylation sites, Asn58, Asn98, Asn185, Asn309 and Asn501, but the last of these, Asn501, was found to be unoccupied (Brumshtein et al. 2010; Limkul et al. 2016). GCase transiently expressed in the WT and mutant lines was purified using two‐step purification with a ConA column and Phenyl 650 M column (Figure 8a). In this study, the *N‐*glycan structures on the Asn19, Asn59, Asn146 and Asn270 sites of the trypsin‐digested *N*‐glycopeptides were identified by nanoLC–MS/MS analysis (Figure 8b, Table 3 and Figure S6). All four *N*‐glycopeptides of GCase produced in the WT and *alg3* plants were modified with 100% plant‐specific *N*‐glycans, of which either M3FX or one of the structures GN2M3FX and GNM3FX was the major *N‐*glycan. On the other hand, the GCase produced in *gntI* mutant contained exclusively mannosidic *N‐*glycans, with M5 as the predominant structure (62.9%–100%) across the *N*‐glycosylation sites. Similarly, in the *alg3gntI* mutant, all four *N*‐glycosylation sites of the GCase were occupied with M3, which accounted for 55.7%–100% of total *N‐*glycans. Interestingly, the immature glucosylated *N*‐glycans detected in Varlilumab were not found in GCase. This result suggests that the same *N*‐glycosylation machinery could function slightly differently depending on the target protein. In particular, the *gntI* mutant producing predominantly M5 *N*‐glycosylated GCase showed its potential to function as an expression host for lysosomal enzymes.

**TABLE 3:** *N*‐glycosylation compositions of the GCase *N*‐glycopeptides.

	Structure		Ratio (%)															
			Asn58				Asn98				Asn185				Asn309			
	Composition	Abbreviation	WT	*alg3*	*gntI*	*alg3gntI*	WT	*alg3*	*gntI*	*alg3gntI*	WT	*alg3*	*gntI*	*alg3gntI*	WT	*alg3*	*gntI*	*alg3gntI*
Mannosidic type	Hex_1_HexNAc_2_	M1	—	—	—	—	—	—	—	—	—	—	—	—	—	—	—	10.3
	Hex_2_HexNAc_2_	M2	—	—	—	—	—	—	—	—	—	—	—	20.4	—	—	—	34.0
	Hex_3_HexNAc_2_	M3	—	—	—	100	—	—	—	100	—	—	—	79.6	—	—	11.9	55.7
	Hex_4_HexNAc_2_	M4	—	—	—	—	—	—	12.7	—	—	—	34.4	—	—	—	25.3	—
	Hex_5_HexNAc_2_	M5	—	—	100	—	—	—	87.3	—	—	—	65.6	—	—	—	62.9	—
**Plant‐specific type**																		
	DeoxyHex_1_HexNAc_2_	F	—	—	—	—	—	—	—	—	—	—	—	—	—	1.0	—	—
	Hex_2_DeoxyHex_1_HexNAc_2_	M2F	—	—	—	—	—	—	—	—	—	—	—	—	3.2	4.7	—	—
	Hex_3_DeoxyHex_1_HexNAc_2_	M3F	—	—	—	—	—	—	—	—	—	—	—	—	—	1.6	—	—
	Hex_1_DeoxyHex_1_Pent_1_HexNAc_2_	MFX	—	—	—	—	—	—	—	—	—	—	—	—	7.3	6.3	—	—
	Hex_2_DeoxyHex_1_Pent_1_HexNAc_2_	M2FX	—	—	—	—	—	—	—	—	3.8	—	—	—	8.7	7.4	—	—
	Hex_3_DeoxyHex_1_Pent_1_HexNAc_2_	M3FX	34.2	59.7	—	—	32.6	100	—	—	9.6	36.1	—	—	10.2	14.7	—	—
Terminal GlcNAc type	Hex_2_Pent_1_HexNAc_3_	GNM2X	—	—	—	—	—	—	—	—	3.0	16.5	—	—	—	—	—	—
	Hex_3_Pent_1_HexNAc_4_	GN2M3X	—	—	—	—	—	—	—	—	—	—	—	—	13.9	20.9	—	—
	Hex_2_DeoxyHex_1_Pent_1_HexNAc_3_	GNM2FX	—	—	—	—	—	—	—	—	—	—	—	—	7.3	6.0	—	—
	Hex_3_DeoxyHex_1_Pent_1_HexNAc_3_	GNM3FX	33.4	40.3	—	—	17.4	—	—	—	31.0	17.2	—	—	15.9	13.2	—	—
	Hex_3_DeoxyHex_1_Pent_1_HexNAc_4_	GN2M3FX	32.4	—	—	—	50.0	—	—	—	49.5	30.2	—	—	31.4	20.6	—	—
Galactosylated type	Hex_4_DeoxyHex_1_Pent_1_HexNAc_4_	GalGN2M3FX	—	—	—	—	—	—	—	—	—	—	—	—	—	1.2	—	—
	Hex_4_DeoxyHex_2_Pent_1_HexNAc_4_	GalFGN2M3FX	—	—	—	—	—	—	—	—	3.1	—	—	—	2.2	2.4	—	—
**Total**																		
Mannosidic type			—	—	100	100	—	—	100	100	—	—	100	100	—	—	100	100
Plant–specific type			100	100	—	—	100	100	—	—	100	100	—	—	100	100	—	—

## 3 Discussion

Over the past two decades, studies deciphering the plant *N*‐glycosylation pathway have provided us with a comprehensive understanding of the *N*‐glycosylation process. The identification of numerous glycosyltransferases, glycosylhydrolases, and other related enzymes has revealed striking similarities between plant and mammalian *N*‐glycosylation pathways, despite some key differences in later processing steps in Golgi (Strasser et al. 2021). Along with the growing interest in the use of plants for biopharmaceutical production, numerous studies have focused on engineering plant *N*‐glycosylation pathways to eliminate plant‐specific epitopes and produce proteins with more humanised *N*‐glycan structures (Castilho et al. 2011; Castilho and Steinkellner 2012; Jansing et al. 2019; Kallolimath et al. 2016). More recently, CRISPR/Cas9 technology has been utilised to precisely modify *N*‐glycosylation related genes in various plant expression systems, further enhancing the potential of plants as biofactories for recombinant *N*‐glycoproteins with desired *N*‐glycan profiles (Kao et al. 2024). The first plant‐produced pharmaceutical approved by the FDA was Elelyso, a recombinant GCase expressed in carrot suspension cell culture (Shaaltiel et al. 2007). The development of Elelyso demonstrated the potential of plant‐based enzyme replacement therapy for lysosomal storage diseases. Since then, several studies have been conducted to meet the *N*‐glycan requirements of lysosomal enzymes using *N*‐glycoengineering approaches. For example, α‐L‐iduronidase has been expressed in the seeds of *A. thaliana* *cgl* mutant (Zeng et al. 2019) and in *A. thaliana cgl* and *gm1* mutants (Pierce et al. 2017). Human acid α‐glucosidase was expressed in ⍺‐mannosidase I mutant rice cell culture and the predominant *N*‐glycan structures were M8 (63.8%) (Jung 2022). GCase, which was stably expressed in RNAi induced *GNTI*‐knockdown *N. benthamiana* plants, contained mostly M5 (ranging from 48.5% to 73% of the total *N*‐glycans between four *N*‐glycosylation sites) (Limkul et al. 2016). When GCase was transiently expressed in the same platform, the M5 content was higher (ranging from 73% to 89.2% of total *N*‐glycans) (Uthailak et al. 2021). These studies provided a valuable approach for enhancing the mannosidic *N*‐glycosylation in *N. benthamiana*. However, the silencing efficiency of RNAi could vary between generations (Kusaba 2004).

In this study, focusing on the homogeneous mannosidic‐type *N*‐glycans on recombinant proteins, we first identified the putative *NbALG3* and *NbGNTI*s genes, encoding key enzymes involved in the ER *N*‐glycan synthesis and Golgi *N*‐glycan maturation, respectively, and characterised their enzymatic properties. *N. benthamiana* possessed single active ALG3 and GNTI enzymes, although GNTI had a nonfunctional pseudoenzyme, NbGNTI‐B. *N. benthamiana* has an allotetraploid genome derived from two diploid ancestral genomes. However, the presence of only a single functional gene locus for certain genes is not uncommon in allotetraploid plants and can be explained by the evolutionary loss or silencing of duplicate genes to balance gene dosage and maintain cellular function (Freeling 2009). To produce recombinant proteins with mannosylated and quality‐controlled uniform *N*‐glycans without plant‐specific epitopes in *N. benthamiana*, single‐ and double‐mutant plants were generated by targeting *NbALG3* and *NbGNTI*s using the multiplex CRISPR/Cas9 genome editing tool. In our study, knockout of *NbGNTIs* in *N. benthamiana* resulted in the highly uniform M5 *N*‐glycan structure with complete elimination of plant‐specific epitopes, consistent with findings in *A. thaliana* where *GNTI* knockout resulted in the absence of β1,2‐xylose and α1,3‐fucose epitopes (Strasser et al. 2005). In this study, the *alg3gntI* double mutant exhibited highly uniform M3 *N*‐glycan structures with complete elimination of plant‐specific epitopes. Notable underglycosylation of the Varlilumab heavy chains was observed in the *alg3gntI* mutant. Additionally, significant phenotypic abnormalities were observed, including impaired root development, dwarf morphology and compromised seed maturation. The deletion of the *ALG3* gene in *S. cerevisiae (Δalg3)* has been consistently reported to cause underglycosylation of proteins across multiple studies due to reduced oligosaccharyltransferase (OST) efficiency toward the altered *N*‐glycan structure (Aebi et al. 1996; Bailey et al. 2012; Parsaie et al. 2013). A previous study reported that a double mutation of *cglalg3* in *A. thaliana* resulted in a profile in which M3 accounted for 98.8% of total *N*‐glycan, but the phenotype of the plant was not mentioned (Henquet et al. 2008). The same root growth defect observed in our study was also reported by another group in the *A. thaliana* g*ntIalg3* mutant (Veit et al. 2018). Previous studies have demonstrated that disrupted *N*‐glycosylation in *A. thaliana* , caused by the loss of specific OST subunits, leads to multiple physiological defects including impaired growth, abnormal cell differentiation, reproductive failure due to inhibited pollen release, and reduced tolerance to salt and osmotic stress (Farid et al. 2013; Hoeberichts et al. 2008; Jeong et al. 2018; Koiwa et al. 2003; Lerouxel et al. 2005; Müller et al. 2016). In *S. cerevisiae* , the terminal α1,2‐glucose residues of Glc3M9‐PP‐Dol were the key element for OST substrate recognition (Burda and Aebi 1998). *N. benthamiana alg3*, which theoretically has the same Glc3M5^ER^‐PP‐Dol substrate for the OST complex as *alg3gntI*, produced a lower amount of underglycosylated Varlilumab heavy chain than *alg3gntI* (Table 2). This result shows that the phenotypic abnormalities of *alg3gntI* were caused not only by reduced compatibility between the Glc3M5^ER^‐PP‐Dol substrate and OST complex, but also by other contributing factors.

It is likely that these phenotypic abnormalities in *N. benthamiana alg3gntI* partly result from underglycosylation of proteins due to reduced OST complex efficiency. Ultimately, the accumulation of non‐glycosylated proteins in the ER triggered the ER stress and activated the unfolded protein response (UPR) (Chen et al. 2023). In *A. thaliana* , chronic ER stress induced by loss of BONI‐associated protein 2 (BAP2) resulted in higher H_2_O_2_ concentrations in the cells, leading to programmed cell death. Additionally, the *bap2* mutant showed significantly lower shoot weight and chlorophyll content compared to the Col‐0 mutant (Pastor‐Cantizano et al. 2024). In our study, ascorbic acid supplementation partially rescued the root growth in *N. benthamiana alg3gntI* (Figure 6d). As a potent antioxidant, ascorbic acid indirectly alleviated some ER stress and improved the cellular function despite the underlying *N*‐glycosylation defect in *N. benthamiana*. However, the same ascorbic acid supplementation did not affect the impaired root growth in *A. thaliana cglalg3* (Figure S5d). *Arabidopsis* GnTII mutants lacking specific GlcNAc residues exhibited increased sensitivity to stress conditions (in the presence of tunicamycin, NaCl, etc.), accelerated leaf senescence, and altered phytohormone responses (particularly to cytokinins and auxin transport inhibitors) (Yoo et al. 2021). While many studies have indicated the importance of complex plant‐specific *N*‐glycosylation to the physiology of certain plant models, the underlying mechanism between the specific type of *N*‐glycans and the defective phenotype is still not clear. For instance, the lack of plant‐specific *N*‐glycan in *A. thaliana* and *N. benthamiana* did not cause noticeable phenotypic changes (Jansing et al. 2019; Strasser et al. 2004), while the same modification in *Oryza sativa* (rice) resulted in severe developmental defects (Fanata et al. 2013; Jung et al. 2021). Phenotypic changes resulting from *N*‐glycosylation modifications vary across plant species, and thus, warrant thorough investigation. Our findings provide some insights into how the knockout of the genes encoding ALG3 and GNTI enzymes affects the growth pattern and root development in *N. benthamiana*.

Interestingly, the glucosylated immature *N*‐glycans detected in Varlilumab, which is expressed in the *alg3* and *alg3gntI*, were not found in GCase. We assume these aberrant *N*‐glycosylations were the result of inefficient ER to Golgi trafficking caused by either ER stress or reduced affinity to the cargo receptors. However, the plant ER to Golgi trafficking remains insufficiently studied, and whether the underlying mechanism is vesicle‐based or tubular remains a matter of some controversy (Brandizzi 2018; Fougère et al. 2025; Robinson et al. 2015). In the mammalian system, the ER‐Golgi intermediate compartment (ERGIC) plays the main role in the trafficking of *N*‐glycoproteins. In cargo receptors such as ERGIC‐53, a lectin binds to high‐mannose‐type *N*‐glycans on the *N*‐glycoprotein and mediates the transportation (Appenzeller et al. 1999; Zhang et al. 2023). The reason why the glucosylated immature glycans were not detected in the GCase might be related to the four *N*‐glycosylation sites increasing the efficiency of transport from the ER to Golgi. On the other hand, *N‐*glycans in the Fc region of IgG are structurally buried between the two CH2 domains (Russell et al. 2018). This structural shielding potentially limits access by ER‐resident α‐glucosidase II after proper folding. This unique spatial feature of antibody *N*‐glycans may explain the presence of GlcM5^ER^ and Glc2M5^ER^ structures in Varlilumab.

NanoLC‐MS/MS analysis revealed that the GCase produced in *gntI* mutant had an *N*‐glycan structure of M5 on all four *N*‐glycosylation sites. These results highlight the promising potential of *N. benthamiana gntI* as a production platform for lysosomal enzymes used in enzyme replacement therapy. To date, numerous studies have focused on *N*‐glycoengineering diverse expression hosts—ranging from yeast to HEK293 cell lines—to produce lysosomal enzymes bearing high‐mannose‐type *N*‐glycans (Jin et al. 2018; Jung 2022; Limkul et al. 2016; Uthailak et al. 2021). Such high‐mannose *N*‐glycosylation is essential for proper lysosomal targeting via the mannose‐6‐phosphate receptor pathway (Kornfeld 1987), making this plant‐based system particularly valuable for therapeutic protein production. Human recombinant α‐L‐iduronidase produced in *A. thaliana* *cgl* seeds contained predominant M5 *N‐*glycan and had enzymatic activities similar to a commercial recombinant enzyme produced in Chinese hamster ovary cells for the treatment of mucopolysaccharidosis I, a lysosomal storage disease. The purified enzyme was subjected to in vitro addition of a mannose‐6‐phosphate tag (He et al. 2013). Early *N*‐glycoengineering research demonstrated that GCase chemically coupled with synthetic trimannosyldilysine (Man_3_Lys_2_) exhibited 11‐fold higher affinity for receptor‐mediated uptake in rat alveolar macrophages compared to the native enzyme (Doebber et al. 1982). *N. benthamiana alg3gntI* mutant, which yields predominantly uniform M3 *N*‐glycans, may serve as a useful platform for lysosomal enzyme production. However, phenotypic abnormalities observed in the mutant could pose limitations. Further optimisation and development of suspension cell culture derived from this mutant may help address the challenges. In contrast, *N. benthamiana gntI* and *alg3gntI* mutants generated in this study are not suitable hosts for the production of therapeutic antibodies. Antibodies produced in these lines predominantly carried mannosidic *N*‐glycans, which are associated with reduced serum half‐lives due to rapid clearance by mannose receptors on macrophages (Boune et al. 2020; Goetze et al. 2011). Despite this limitation, we employed Varlilumab as a model protein to investigate how these mutations influence *N*‐glycosylation machinery in a various protein context.

In this study, we have comprehensively characterised the *N*‐glycosylation machinery of *N. benthamiana alg3*, *gntI* and *alg3gntI mutants*, elucidating their distinctive phenotypes and highlighting their promising applications and drawbacks for the production of biopharmaceuticals. In particular, *gntI* and *alg3gntI* mutants, which produce highly uniform mannosidic structures, could serve as alternative expression platforms for producing pharmaceutical proteins that require mannosidic *N*‐glycans, especially after further optimisation.

## 4 Experimental Procedures

### 4.1 Materials

The 2‐aminopyridine (PA)‐labelled oligosaccharides, PA‐Sugar chains, were purchased from TaKaRa Bio (Shiga, Japan) and Masuda Chemical Industries (Tokushima, Japan) or prepared in‐house using the *Arabidopsis N*‐glycosylation mutant line as described later. The following yeast strains were used in this study: YG170 (*MATa ade2‐101 ade3 ura3‐52 his3 alg3‐1 stt3‐3*) and YG176 (*MATa ade2‐101 ade3 ura3‐52 his3Δ200 leu2 tyr1 stt3‐3*) (Zufferey et al. 1995).

### 4.2 In Vivo Assay of NbALG3

Total RNA was extracted from *N. benthamiana* leaves using the QIAGEN RNeasy Plant Mini Kit (QIAGEN, Chatsworth, CA) and treated with DNase (Promega, Madison, WI) to remove genomic DNA contamination. Subsequently, 1.0 μg of RNA was used for reverse transcription with a SuperScript VILO cDNA Synthesis Kit (Invitrogen, Carlsbad, CA). The open reading frame of *NbALG3* was cloned using cDNA prepared from 5‐week‐old *N. benthamiana* leaves and ligated into the pYES2 vector (Invitrogen). The resultant vector was introduced into the *S. cerevisiae* YG170 strain by electroporation as previously described (Kajiura et al. 2010). The growth assay and lipid‐linked oligosaccharide analysis were also performed as described previously (Kajiura et al. 2010). The HPLC to separate PA‐labelled lipid‐linked oligosaccharides was performed using the mobile phase composed of acetonitrile/acetic acid (solvent A: 98/2, v/v) and water/acetic acid/triethylamine (solvent B: 92/5/3, v/v/v). The PA‐sugar chain was separated using an InertSustainNH2 column (2.1 mm ID × 150 mm; GL Sciences Inc., Tokyo, Japan) by linearly increasing the solvent B concentration from 20% to 55% over 35 min at a flow rate of 0.2 mL/min. Fluorescence intensity was measured to monitor the eluted fractions at excitation and emission wavelengths of 310 and 380 nm, respectively. The molecular masses of the target peak were analysed by LC‐MS/MS (Kajiura et al. 2015).

### 4.3 Heterologous Expression, Purification and Activity Assay of Recombinant NbGNTI

A cDNA fragment encoding NbGNTIs (amino acids 81–442 for NbGNTI‐A and 81–400 for NbGNTI‐B) excluding a putative transmembrane domain was prepared using *N. benthamiana* cDNA as a template. The PCR products were ligated into pCold I vector (TaKaRa Bio). The resultant vector was introduced into the *E. coli* BL21(DE3) strain by electroporation.

The cells were initially cultured in 2 mL of 2× YT medium (16 g/L tryptone, 10 g/L yeast extract and 5 g/L NaCl) supplemented with 50 μg/mL ampicillin at 37°C for 12–14 h. Subsequently, 2 mL of this preculture was transferred into 200 mL of fresh medium and incubated until the OD_600_ reached 0.5. Protein expression was induced by adding 1 mM IPTG, followed by incubation at 15°C for 20 h. The recombinant proteins were purified using a Co^2+^ affinity column (Clontech, TaKaRa Bio) with the elution buffer containing 500 mM imidazole in equilibrium buffer. The eluate buffer was exchanged using an Amicon Ultra Centrifugal device (Merck Millipore, Burlington, MA).

The PA‐labelled oligosaccharide, M5‐PA, was prepared from the *Arabidopsis gntI* mutant, commonly referred to as the *cgl1* mutant (Kang et al. 2008; von Schaewen et al. 1993), as previously reported (Kajiura et al. 2010). The enzyme assay was performed using a mixture of 20 mM cacodylic acid buffer (pH 6.5), 10 mM MnCI_2_, 5 mM UDP‐GlcNAc, 10 pmol of a PA‐sugar chain as a substrate, and recombinant NbGNTI at 30°C for 1 h. The reaction was stopped by boiling, and the mixture was centrifuged at 12 000 × *g*. The obtained supernatants were subjected to HPLC. The mobile phase consisted of water/acetonitrile with 0.02% trifluoroacetic acid (Solvent A: 100/0 v/v; Solvent B: 80/20 v/v). HPLC was carried out on a Cosmosil 5C18‐AR‐II column (4.6 × 250 mm; Nacalai Tesque, Kyoto, Japan) using a HITACHI LaChrom HPLC System. The concentration of Solvent B was linearly increased from 0% to 20% over 35 min at a flow rate of 0.7 mL/min. The fluorescence intensity was the same as described above. The peaks of interest were collected, and the molecular masses of the reaction products were analysed by MALDI‐TOF MS using an autoflex speed mass spectrometer (Bruker, Bremen, Germany).

### 4.4 The Sequence Analysis of Genomic DNA

The sequence containing the region targeted for genome editing using CRISPR/Cas9 was amplified using genomic DNA as a template by PCR; the product was sequenced by Sanger sequencing. Genomic DNA was extracted following the procedure described below. Based on the sequence, gRNAs were designed, and a plant genome editing vector was constructed.

### 4.5 Plasmid Construction of the Multiplex CRISPR/Cas9 Targeting *ALG3* and *GNTI* Genes

The multiplex CRISPR/Cas9 plasmid was constructed. Two sets of gRNA were designed for each gene and inserted into pBS‐based gRNA plasmids separately. All four gRNA cassettes were incorporated into pWAT‐135‐FLAG‐hcoCas9 × 5‐cc‐smGFPER by Golden Gate Cloning using a NEBridge Golden Gate Assembly Kit (NEB). *E. coli* DH5α was transformed by the reaction mix. The positive colonies were checked by PCR and the plasmid sequence was confirmed by Sanger sequencing.

### 4.6 CRISPR/Cas9‐Mediated *alg3gntI* Transformation of *N. benthamiana*

The leaves of the WT *N. benthamiana* grown in a sterile pot were cut into 5 mm^2^ discs and co‐incubated with *Agrobacterium tumefaciens* GV3101 harbouring pWAT135‐Cas9‐NbALG3GNTI for 3 days. The leaves were then transferred to a callus‐inducing Murashige and Skoog (MS) plate supplemented with 2 μg/mL NAA, 0.2 μg/mL BA and 250 μg/mL carbenicillin. Callus was induced for 14 days and then the calluses were transferred to a plate containing shoot‐inducing MS medium supplemented with 0.1 mg/L NAA, 1 mg/L BA, 250 mg/L carbenicillin and kanamycin. The concentration of the selection antibiotic, kanamycin, was increased from 50 to 75 to 100 μg/mL at two‐week intervals. The developed shoots were then moved to a medium without hormones containing 100 μg/mL kanamycin for root development. Transformant plantlets were transferred to soil when the root and leaf size were sufficient.

### 4.7 Genomic DNA Extraction From Plant Leaves

Genomic DNA (gDNA) was extracted as described in (Chabi et al. 2015) with some modifications. Briefly, a 1 cm^2^ piece of fresh leaf tissue was homogenised in liquid nitrogen, and 200 μL Genomic DNA extraction buffer (200 mM Tris–HCl pH 7.5, 250 mM NaCl, 25 mM EDTA pH 8.0, 0.5% SDS) was added and gently mixed. The sample was incubated for 10 min at RT and centrifuged at 10 000 × *g* for 10 min at RT. The supernatant was transferred to a new tube, and gDNA was precipitated by isopropanol followed by centrifugation. The extracted gDNA pellets were dried and dissolved in 50 μL dH_2_O.

### 4.8 Sequencing of the *ALG3* and *GNTI* Genes of the T_0_ , T_1_ and T_2_ Mutant *N. benthamiana* Plants

The first round of screening was done by sequencing of the gRNA target regions of the *ALG3* and *GNTI* genes. The target gene amplification was done using KOD‐Plus‐Neo Polymerase (TOYOBO, Osaka, Japan). Amplified products were separated on 1% agarose gel, and the target DNA product was purified from the gel by using a Gel Advanced Gel extraction system (Viogene, Taiwan). The purified DNA product was sequenced by the GeneWiz Sanger sequencing service (Tokyo, Japan).

### 4.9 Generation of the *gntI* Mutant by Cross‐Pollination of the *alg3gntI* Mutant With *N. benthamiana* WT

Cross‐pollination of *N. benthamiana* was performed using a controlled manual pollination approach. Healthy plants were grown in a controlled environment (16 h light/8 h dark cycle at 22°C with 50% humidity) until reaching the flowering stage. One to 2 days prior to pollination, the flowers on designated female parent plants that were about to open but had not yet released pollen were selected for emasculation. Using fine forceps, the petal was carefully opened, and all anthers were removed without damaging the stigma. For pollination, freshly opened flowers from the selected male parent plants with dehiscent anthers were identified. Pollen was collected on sterile cotton swabs by gently touching the anthers and was immediately transferred to the stigma of the emasculated flowers, ensuring complete coverage of the stigmatic surface. To prevent contamination with foreign pollen, pollinated flowers were covered with breathable paper bags. Developing seed pods were monitored regularly and the seeds were harvested. The back‐crossed seeds were grown and screened by anti‐HRP western blotting and target gene sequencing.

### 4.10 Western Blotting and Lectin Blotting to Examine the General *N*‐Glycan Profile

Total soluble proteins were extracted from the 5‐week‐old *N. benthamiana* leaves and digested with the PNGase F (Wako Pure Chemical Industries Ltd., Osaka, Japan) or Endo H_f_ (NEB). The digested protein samples were separated on SuperSep Ace 10%–20% gradient SDS‐PAGE gel (Wako Pure Chemical Industries Ltd.). The proteins were transferred to a polyvinylidene fluoride (PVDF) membrane (Merck Millipore). The membrane was blocked with 0.5% bovine serum albumin in phosphate‐buffered saline with 0.05% Tween20 (PBS‐T) at 4°C overnight. The membrane was transferred to 2 μg/mL biotin‐conjugated ConA in 0.5% BSA containing PBS‐T and incubated for 1 h at RT with shaking. The membrane was washed four times with PBS‐T for 5 min each and incubated with 1:5000 diluted StrepTactin‐HRP in PBS‐T (BioRad) for 1 h at RT.

To detect the plant‐specific *N*‐glycosylation, 1 μg total soluble proteins were blotted to the PVDF membrane as previously described. The membrane was blocked by 0.5% skim milk in PBS‐T at 4°C overnight The membrane was primed with 1:12000 diluted anti‐HRP rabbit antibody in PBS‐T for 1 h at RT. The unbound primary antibodies were removed from the membrane by four times wash with PBS‐T for 5 min each. The membrane was then incubated with 1:12000 diluted HRP‐linked secondary anti‐rabbit IgG (Cytiva, Uppsala, Sweden) in PBS‐T for 1 h at RT. After the final incubation, the membranes were washed four times for 5 min each and Immobilon Classico Western HRP substrate (Merck Millipore) and incubated for 3 min. All the results of the lectin blot and western blot were visualised by iBright CL1500 Imaging system (Invitrogen, Waltham, MA).

### 4.11 Total *N*‐Glycan Analysis of the Mutants

Total *N*‐glycan was extracted from the 5‐week‐old *N. benthamiana* plants and labelled with 2‐aminopyridine (PA) by a previously described method (Kajiura et al. 2010). The PA‐labelled *N*‐glycans were separated by RP‐HPLC using a COSMOSIL 5C18‐AR‐II Packed Column (4.6 mm × 250 mm). The mobile phase consisted of an A buffer of 0.02% trifluoroacetic acid (TFA) in MQ and a B buffer of 0.02% TFA in 20% acetonitrile. The RP‐HPLC run was performed at a flow rate of 0.7 mL/min, and the B buffer gradient was increased from 0% to 20% from 15 min to 50 min. The eluted fractions were monitored by fluorescence intensity at an excitation wavelength of 310 nm and an emission wavelength of 380 nm. All the PA‐labelled *N*‐glycan‐containing elution peaks were collected and lyophilised. The PA‐labelled *N*‐glycan‐containing samples were further separated using an Asahipack NH2P‐50 2D column (2.0 × 150 mm; Showa Denko) and analysed by LC–MS/MS (Kajiura et al. 2015). The ratio of *N‐*glycans was calculated by the peak area in HPLC.

### 4.12 *Agrobacterium*‐Mediated Transient Expression of Model Proteins

The Varlilumab and GCase expression vectors were used for transient expression in *N. benthamiana* (Limkul et al. 2016; Nguyen et al. 2023). All plant expression vectors were transformed into the *A. tumefaciens* LBA4404 strain using a MicroPulser Electroporator (BioRad, Hercules, CA). Transformants were selected on 2 × YT medium containing 50 μg/mL kanamycin, rifampicin and streptomycin. For vacuum agro‐infiltration, a single colony was grown in 5 mL 2 × YT liquid medium containing the antibiotics at 28°C, then scaled up to 200 mL for 2 days. *A. tumefaciens* cells were collected and resuspended in infiltration buffer containing 10 mM MgSO_4_, 10 mM MES, pH 5.8 at an OD_600_ of 0.5. For Varlilumab expression, the P19 suppressor, heavy chain (HC) and light (LC) chain vectors were mixed at a 2:1:1 ratio. For GCase expression, the P19 suppressor and GCase expression vector were mixed at a 1:1 ratio. The soil‐grown, 5‐week‐old WT and *N. benthamiana* mutants (*alg3*, *gntI* and *alg3gntI*) dipped in an infiltration buffer containing *Agrobacterium*, which was prepared earlier, in a vacuum chamber. The vacuum was applied and then the air gauge was opened slowly to ensure the penetration of the leaves by the infiltration buffer. The *Agrobacterium*‐infiltrated plants were placed back in the controlled environment with a 16 h light and 8 h dark cycle at 22°C with 50% humidity. The leaves were harvested after 6 days of infiltration and kept at −80°C until further use.

### 4.13 Varlilumab Purification

The leaf sample was homogenised with a mortar and pestle while adding liquid nitrogen to facilitate plant cell wall disruption. A two‐sample volume of extraction buffer (100 mM sodium dihydrogen phosphate, 100 mM NaCl, 40 mM ascorbic acid pH 7.0) was added to the homogenised leaf sample and vortexed vigorously. Then the sample was incubated on ice for 10 min, followed by vortexing for 5 min, and these steps were repeated. The crude sample was centrifuged at 10 000 × *g* for 20 min. The IgG was purified from the supernatant using a Monospin ProA spin column (GL Sciences Inc.) according to the protocol provided.

### 4.14 GCase Purification

GCase was purified by two‐step purification with an agarose‐bound ConA affinity column (Funakoshi, Tokyo, Japan) followed by a Phenyl 650 M (Tosoh Corp., Tokyo, Japan) hydrophobic interaction column. Both columns were packed and washed with five column volumes (CV) of Milli‐Q water. The ConA column was equilibrated with 5 CV ConA buffer (20 mM Tris–HCl pH 7.0, 50 mM NaCl, 1 mM MgCl_2_, 1 mM MnCl_2_, 1 mM CaCl_2_) and the Phenyl 650 M column was equilibrated with five CV phenyl equilibration buffer (50 mM phosphate buffer pH 7.0, 2 M NaCl) prior to sample preparation.

The leaf sample was homogenised using a mortar and pestle while adding liquid nitrogen; four sample volume extraction buffer (20 mM Tris–HCl pH 7.0, 150 mM NaCl, 0.5% taurocholic acid) was added. Then the sample was vortexed vigorously and incubated on ice for 10 min. The crude sample was centrifuged at 10 000 × *g* for 20 min, and the supernatant was directly applied to a ConA column. The unbound proteins were washed with 15 CV ConA buffer. The GCase was eluted by 10 CV Elution buffer (50 mM PB pH 7.0, 4 mM β‐mercaptoethanol, 1.3 mM EDTA, 0.15% Triton X‐100, 0.125% taurocholic acid and 300 mM Methyl‐α‐D‐mannopyronoside). The NaCl concentration of the ConA elution fraction was adjusted to 2 M and applied to the Phenyl 650 M column. The unbound proteins were washed using five CV phenyl equilibration buffer. GCase was eluted by a series of 50 mM phosphate buffer (pH 7.0)‐based buffers with increased hydrophilicity containing 1.5 M NaCl, 1.0 M NaCl, 0.5 M NaCl, 20% ethylene glycol, 30% ethylene glycol and 50% ethylene glycol. The elution was fractionated by 1 mL/fraction and checked by GCase enzyme activity assay. The fractions exhibiting enzyme activity were pooled and dialyzed against 20 mM Tris pH 7.0 overnight. Dialyzed samples were concentrated by using an Amicon Ultra Centrifugal device (Merck Millipore) according to the instructions provided.

### 4.15 *N*‐Glycan Analysis of the Model Proteins Expressed in WT and Mutant *N. benthamiana* Plants

The purified model proteins were separated by SDS‐PAGE with SuperSep Ace gel (Wako Pure Chemical Industries Ltd.) and stained by CBB stain One Super (Nacalai Tesque). The target band was excised from the gel and sliced into 2 mm^3^ pieces. The gel pieces were destained with 50 mM NH_4_HCO_3_: acetonitrile (1:1, v/v) until the CBB dye was completely removed. The pieces were then dehydrated with pure acetonitrile and rehydrated in trypsin solution containing 10 ng/μL Trypsin gold (Promega), 1% ProteaseMax (Promega) surfactant and 50 mM NH_4_HCO_3_. The trypsin digestion was carried out at 50°C for 1 h. The digested peptide containing the liquid phase was transferred to a new tube, and 0.2 μL TFA was added to stop the digestion.

The nano LC–MS/MS analysis was performed on an ESI‐Qq‐TOF mass spectrometer (micrOTOF‐Q II; Bruker Daltonics) operated with automatic switching between MS and MS/MS modes. The nanoLC system (1200 series; Agilent Technologies, Santa Clara, CA) was equipped with a trap column (5 μm, 0.3 × 5 mm) and an analytical column (3.5 μm, 0.075 × 150 mm), both packed with Zorbax 300SB C‐18 (Agilent Technologies).

### 4.16 Statistical Analysis

Statistical analyses were performed using R Statistical Software Ver. 4.4.3 (R Core Team 2023). Differences between multiple groups were evaluated using one‐way analysis of variance (ANOVA); followed by post hoc testing. For pairwise comparisons, Tukey's honestly significant difference (HSD) test was applied.

## Author Contributions

D.B. performed the generation of the mutant plants, screening, expression and purification of the model proteins, *N*‐glycosylation analysis and writing of the manuscript. H.K. provided supervision and finalisation of the manuscript and conducted *N*‐glycosylation analysis and gene identification experiments. R.L.S.S.‐C. contributed to plant maintenance and agroinfiltration experiments. Y.Y. and A.T. constructed the gRNA and CRISPR/Cas9 expression plasmids. A.T. donated the CRISPR/Cas9 expression plasmid and the P19 RNA silencing suppressor plasmid. T.I. and R.M. provided the technical and revision support. K.F. supervised the project and contributed to the finalisation of the manuscript. All co‐authors read and endorsed the final version of the manuscript.

## Conflicts of Interest

The authors declare no conflicts of interest.

## Supporting information


**Data S1:** pbi70326‐sup‐0001‐Supinfo.docx.

## Data Availability

The original dataset presented in this published article is included in the article and/or Data S1. The data that support the findings of this study are available on request from the corresponding author. The data are not publicly available due to privacy or ethical restrictions.
