# Supplementary material for: CRISPR/Cas9‐Mediated Knockouts of the ALG3 and GNTI in N. benthamiana and Their Application to Pharmaceutical Production
Source: Plant Biotechnol J. 2025 Sep 4;23(12):5894–916. doi: 10.1111/pbi.70326 (PMC12665068; doi:10.1111/pbi.70326)
Supplement: Supplementary file 1 — Data S1: pbi70326‐sup‐0001‐Supinfo.docx. [file PBI-23-5894-s001.docx]

**CRISPR/Cas9-mediated knockouts of the *ALG3* and *GNTI* in *N. benthamiana*and their application to pharmaceutical production**

**Supplementary Information**

**Dolgormaa Bataa, Hiroyuki Kajiura, Reimi Lai Sang Sawada-Choi, Yukino Yamashita, Takeshi Ishimizu, Ryo Misaki, Atsushi Takeda, and Kazuhito Fujiyama**

International Center for Biotechnology, University of Osaka, 2-1 Yamada-oka, Suita-shi, Osaka, 565-0871, Japan

*Correspondence should be addressed to K.F. (fujiyama@icb.osaka-u.ac.jp)


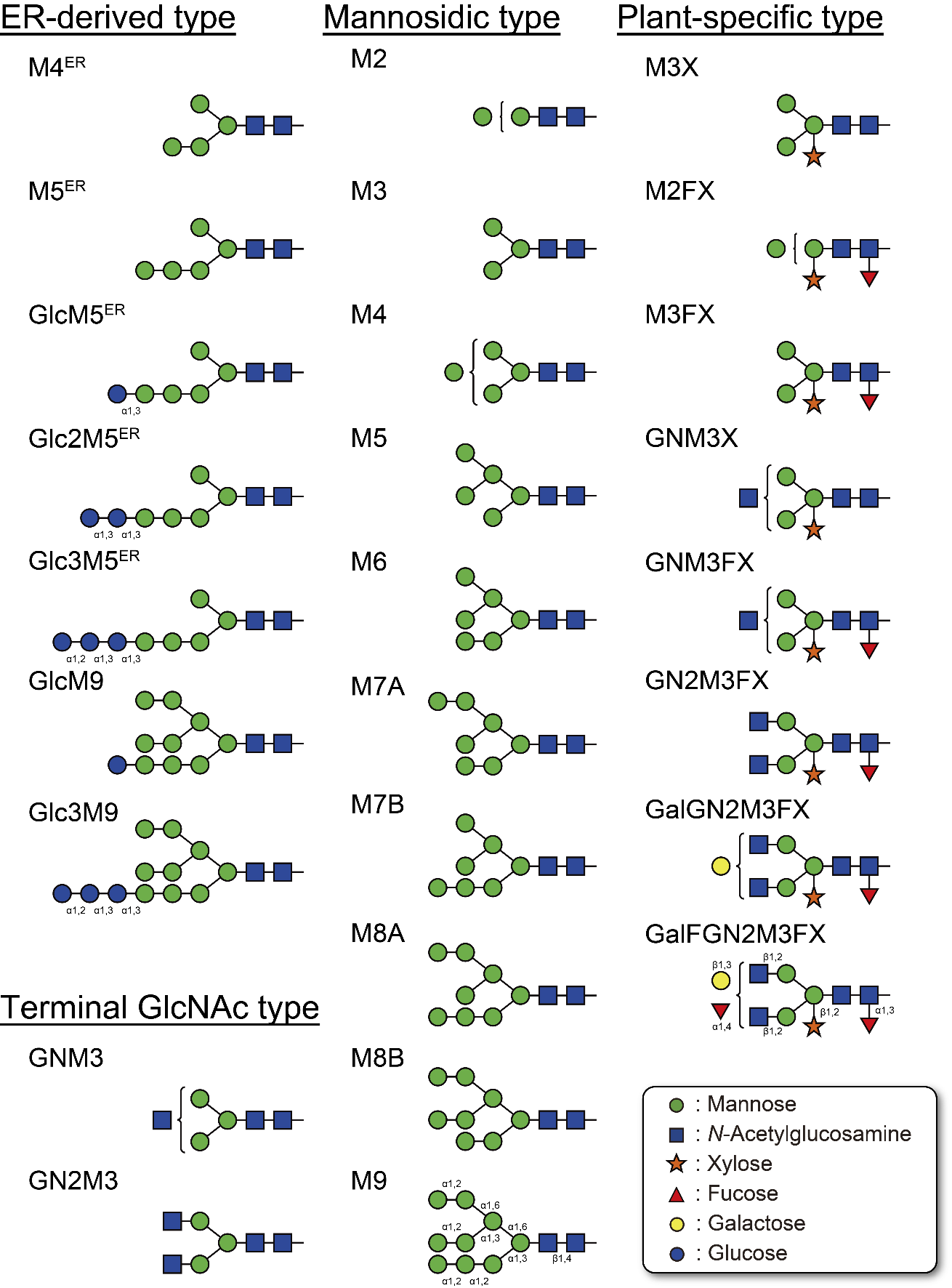


**Supplementary Figure S1 *N*-Glycan abbreviations and their structures.** Specific symbols representing the sugar residues are displayed in the box.

**
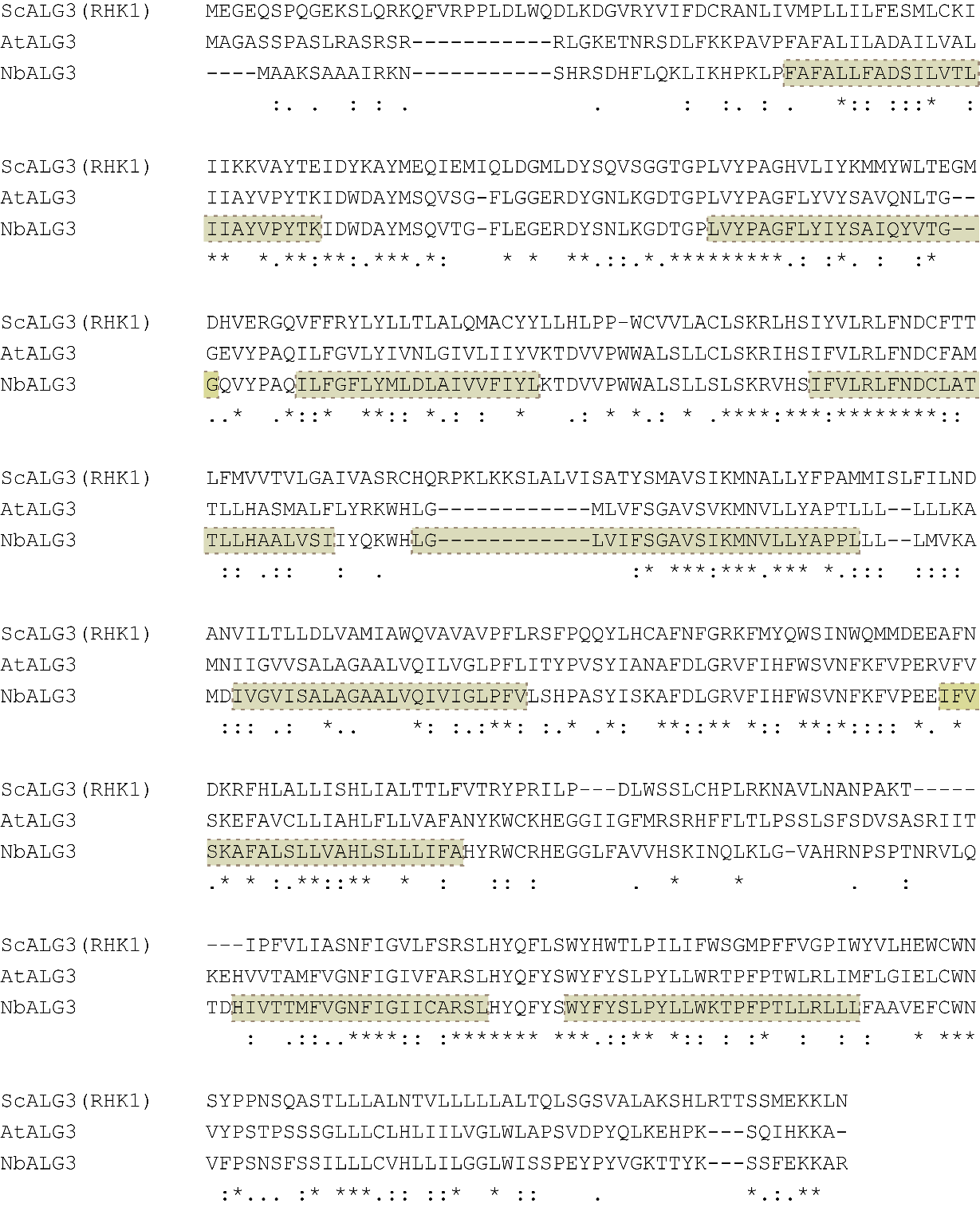
**

**Supplementary Figure S2 Amino acid alignment of ALG3.** The alignment was constructed using *S. cerevisiae* ALG3 (RHK1), Arabidopsis ALG3 (AtALG3, At2g47760), and *Nicotiana benthamiana* ALG3 (NbALG3, Niben101Scf01521g16013.1) using CLUSTALW (http://align.genome.jp/). Similar, conserved substitutions, and identical sequences are indicated by “.”, “:”, and “*”, respectively. Putative transmembrane regions predicted by the TMHMM server are shaded in yellow.


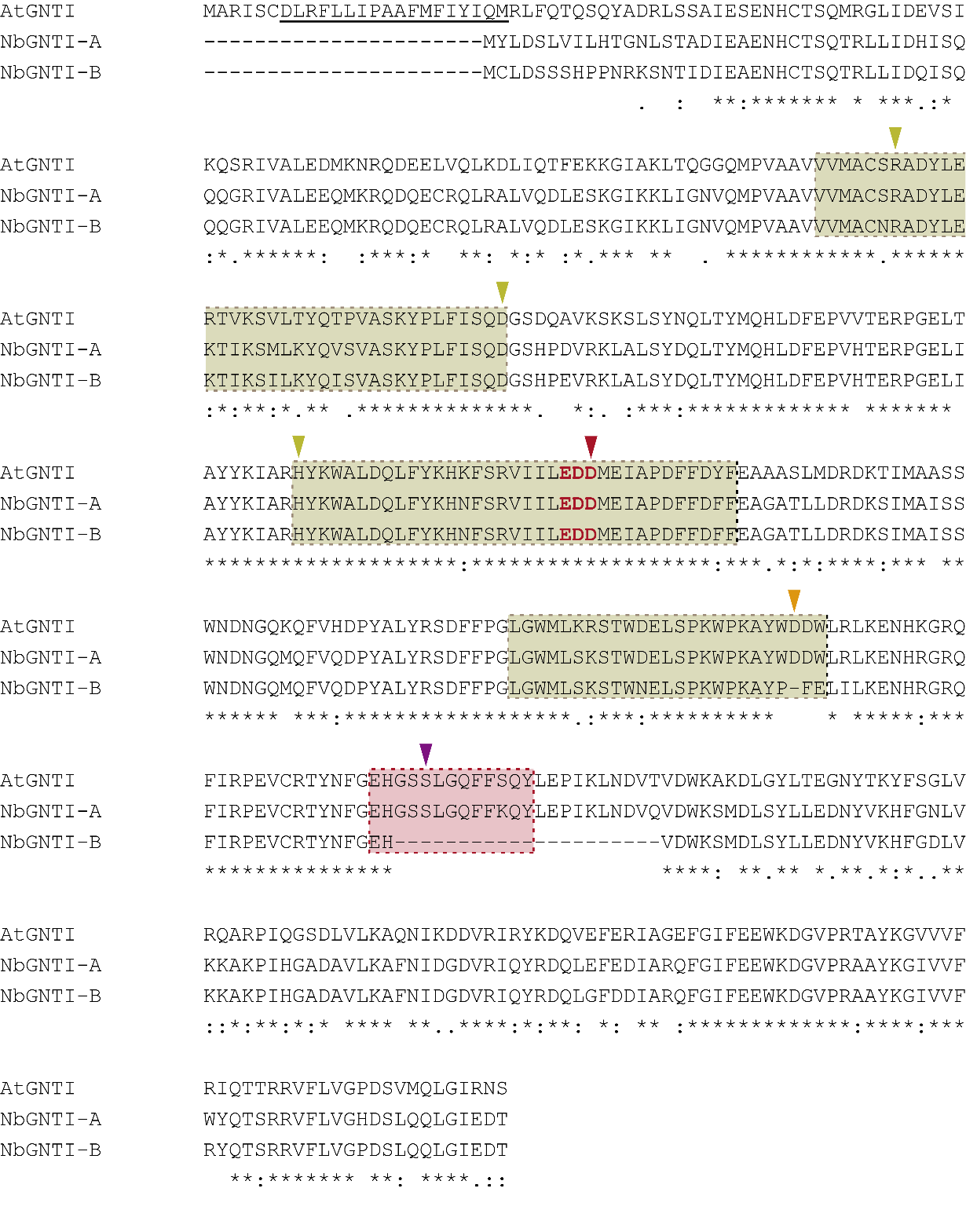


**Supplementary Figure S3 Amino acid alignment of GNTI.** The alignment was constructed using Arabidopsis GNTI (AtGNTI, AT4G38240), and *Nicotiana benthamiana* GNTIs (NbGNTI-A, Niben101Scf04294g06042.1; NbGNTI-B, Niben101Scf01176g01032.1). DxD motif (E^187^DD^189^) essential for Mn^2+^ ion binding and catalysis are shown in bold red. Red, yellow, orange, and purple triangles indicate the direct interaction site with the bound Mn^2+^, the active site for donor substrate binding, the active site for catalysis, and the binding site for UDP-GlcNAc and Mn^2+^ ions, respectively. The conserved sequences among *N*-acetylglucosaminyltransferases proposed by Breton *et al*., and the 13-residue loop essential for UDP-GlcNAc binding are shaded in yellow and red, respectively. “.”, “:”, and “*” shown under the alignment represent similar, conserved substitutions, and identical residues, respectively.


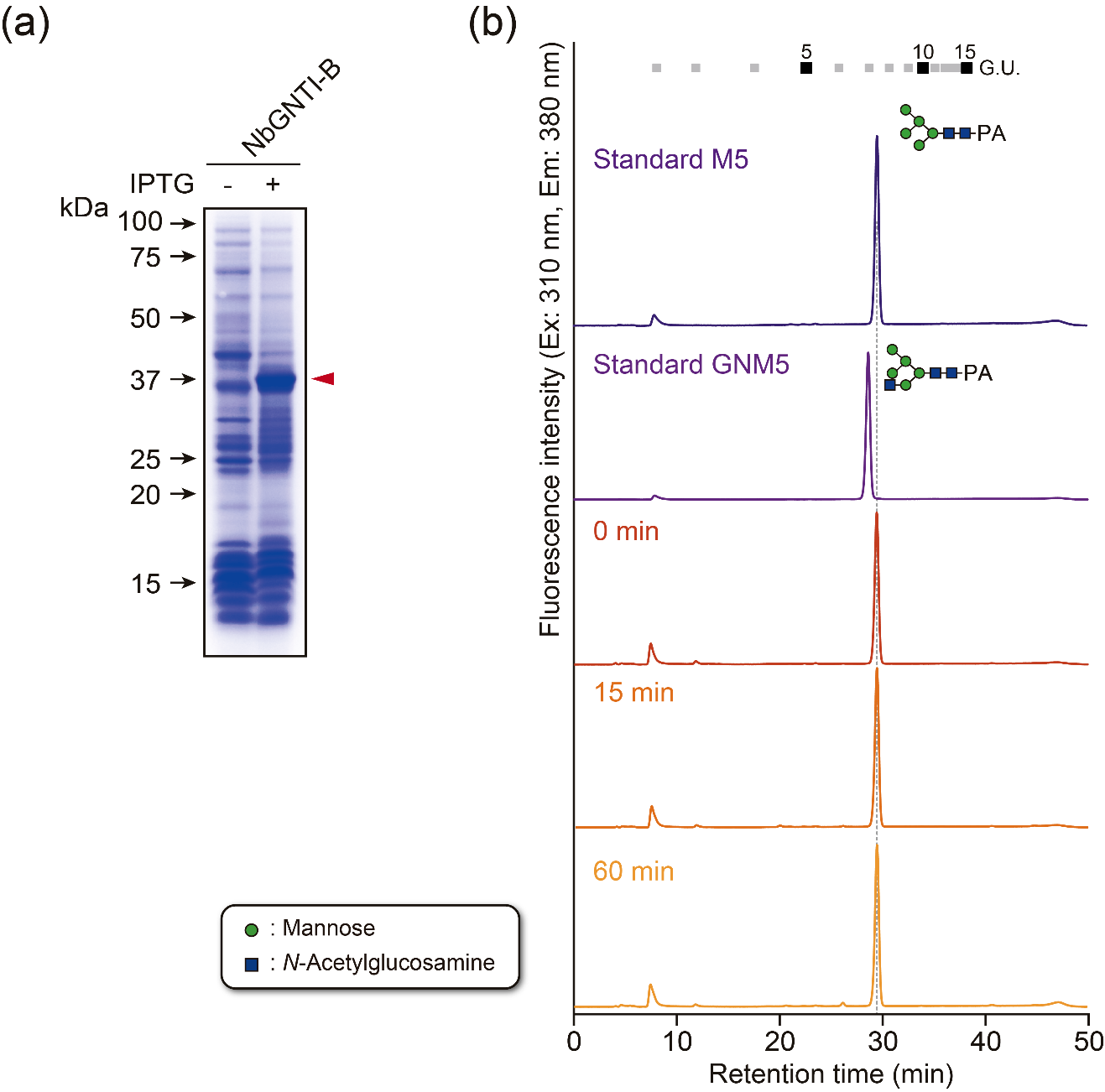


**Supplementary Figure S4 Expression and activity assay of recombinant NbGNTI-B.** (a) CBB staining of recombinant NbGNTI-B. The crude cell lysate of *E. coli* expressing NbGNTI-B was separated by SDS-PAGE and visualized. The red triangle indicates NbGNTI-B. (b) The HPLC profile of the recombinant NbGNTI-B reaction product. The reaction products of NbGNTI-B using Man_5_GlcNAc_2_-PA and UDP-GlcNAc as the acceptor and donor substrates, respectively, were analyzed by RP-HPLC and compared with the authentic PA-sugar chain, GlcNAcMan_5_GlcNAc_2_. Numbers at the top represent the elution positions of glucose units

**
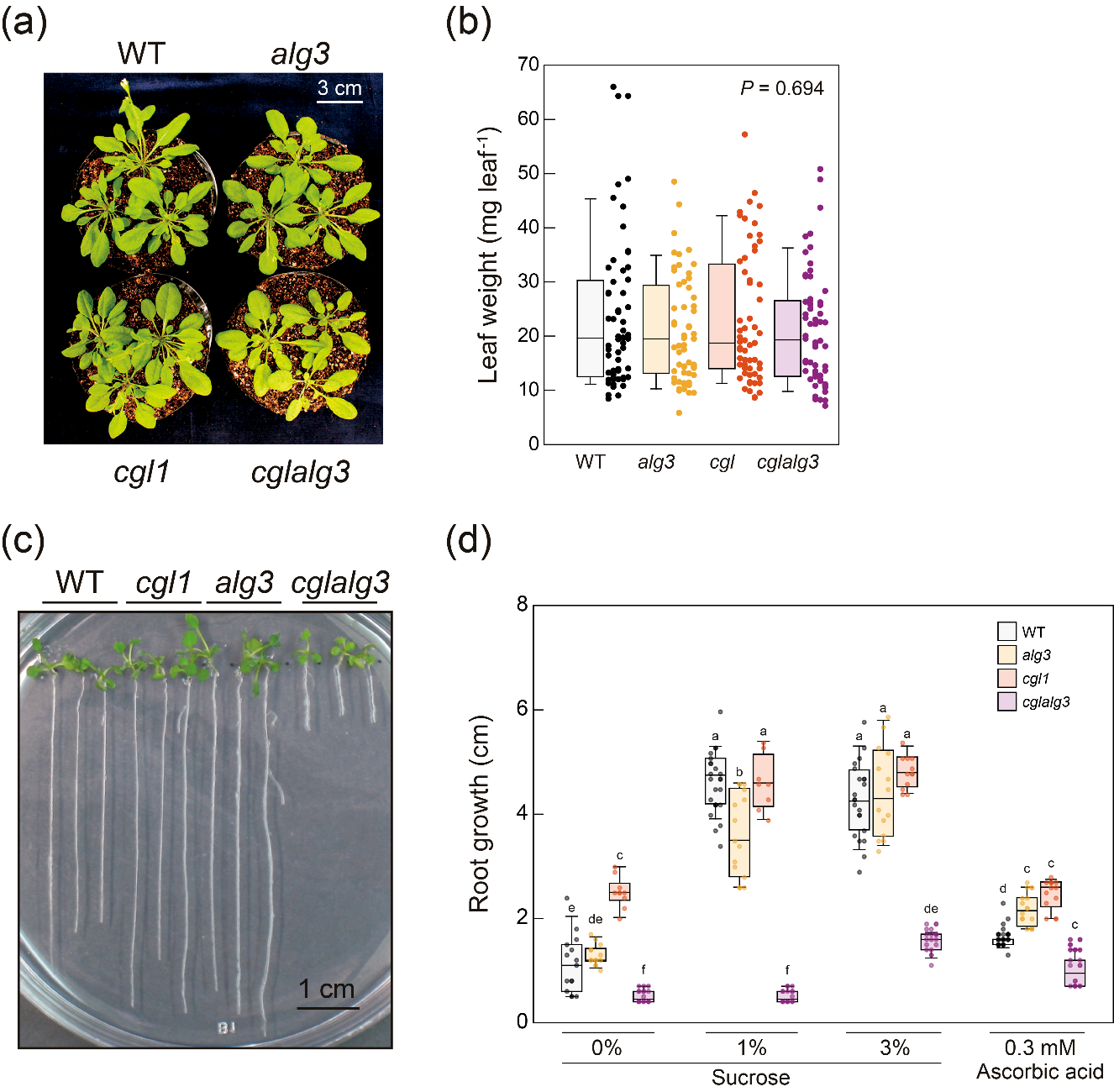
**

**Supplementary Figure S5 Root phenotypic analysis of *A. thaliana* WT, *cgl1*, *alg3* and *cgl1alg3 mutants*.** (a) Top angle image of the *A. thaliana* WT and mutants. (b) Boxplot of fresh leaf weight of the WT and mutant plants; median and individual values were plotted. (c) Root growth comparison of the WT and mutant seedlings. Seven-day-old seedlings on MS plates were transferred to rectangle MS plates with 3% sucrose and grown an additional 10 days. (d) Boxplot of the root length of the WT and mutant seedlings grown and transferred to an MS plate with 0%, 1%, or 3% sucrose and 0.3 mM ascorbic acid supplement; median and individual values are plotted. The letters above each plot represent the significance of the difference between any two groups. A one-way ANOVA was performed to compare the means between groups. The results showed a significant difference among the groups (*p* < 0.05). Tukey’s HSD post-hoc test was conducted for pairwise comparisons.

**
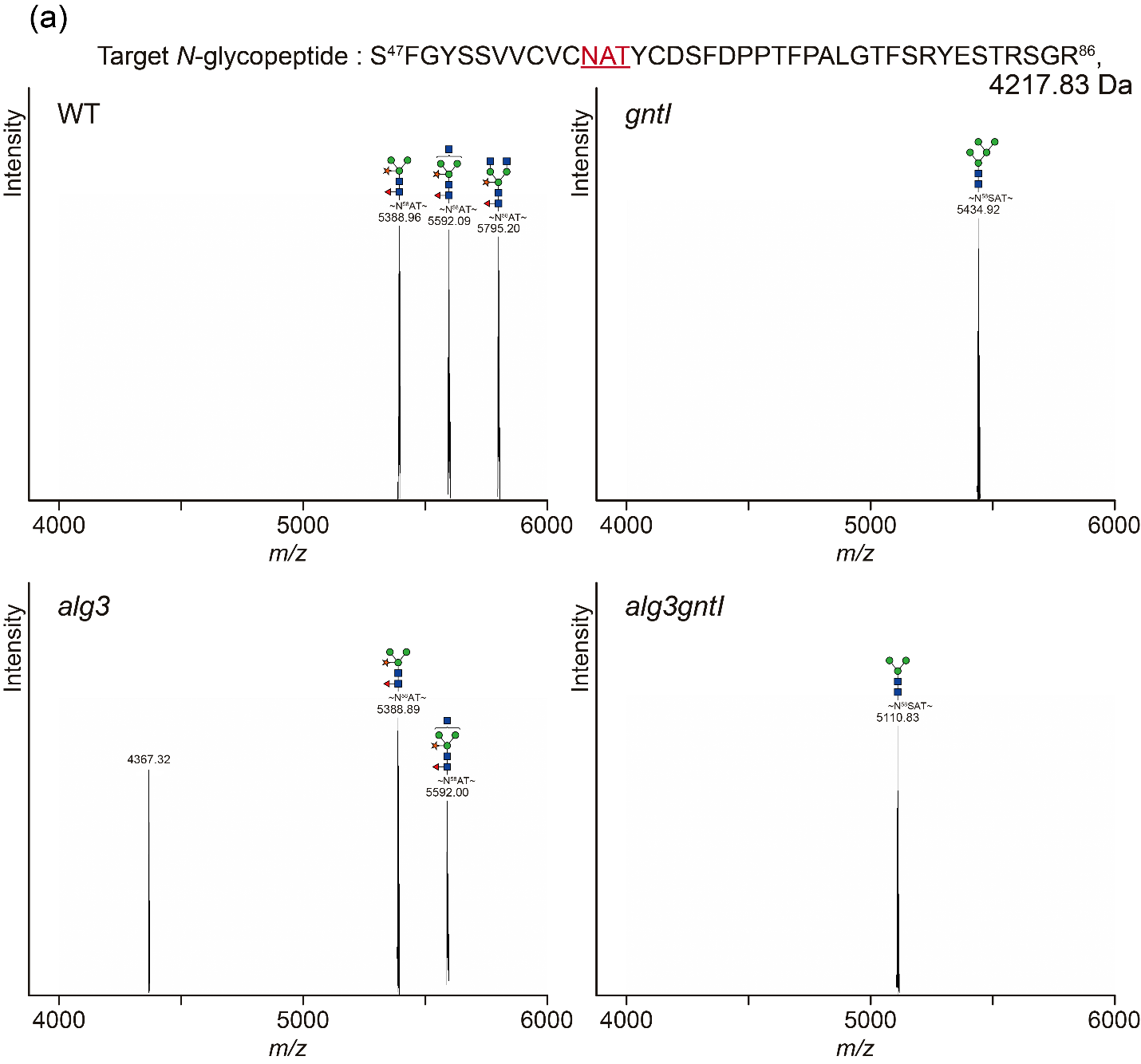
**

**Supplementary Figure S6 *N*-Glycosylation analysis of the other three sites of GCase**. (a) *N*-Glycosylation analysis of Asn58. (b) *N*-Glycosylation analysis of Asn98. (c) *N*-Glycosylation analysis of Asn185. Peptide average masses are shown in parenthesis. All *m/z* signals and corresponding *N*-glycan structures of *N*-glycopeptides are displayed.

**
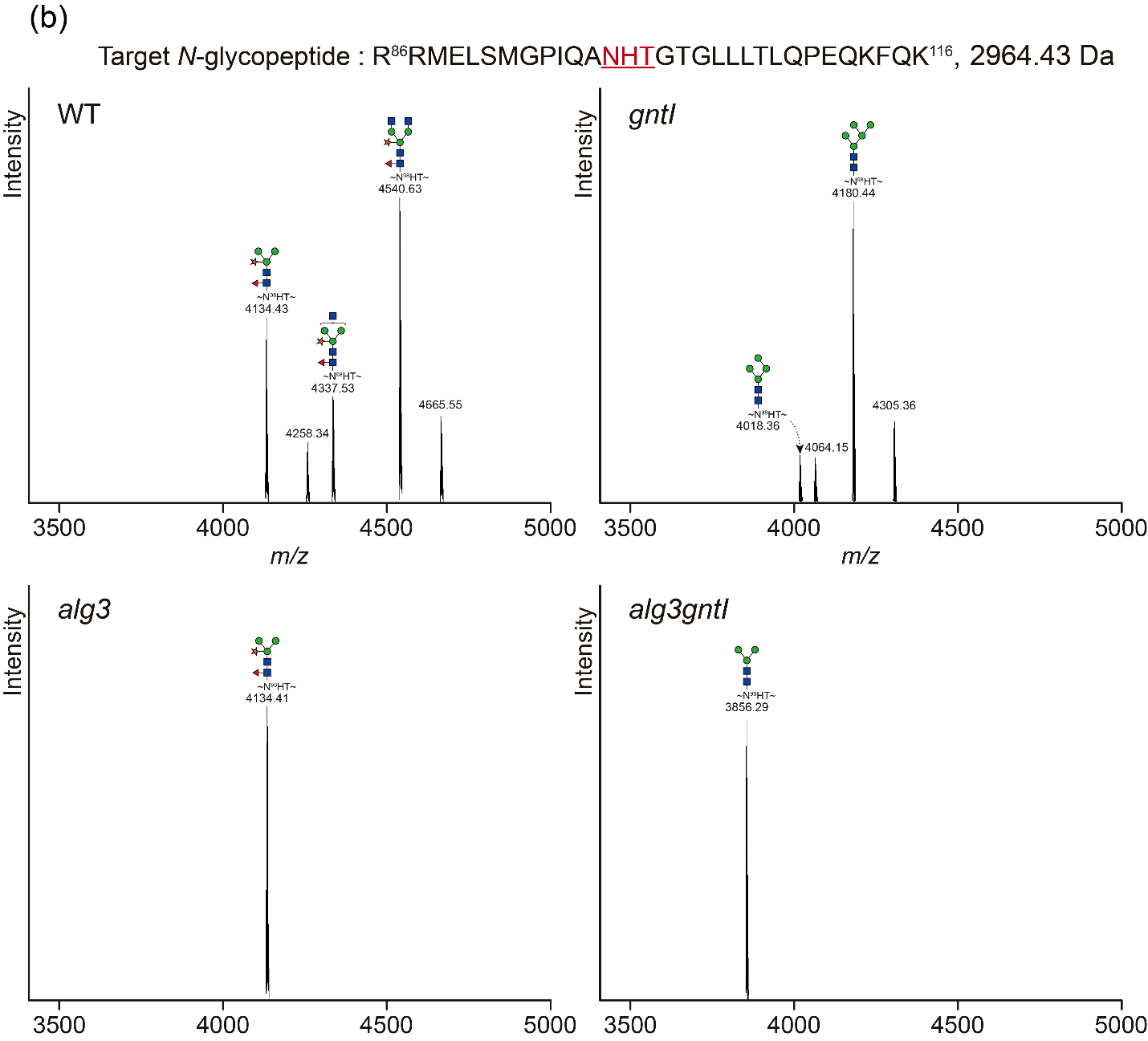
**

**Supplementary Figure S6,** Continued

**
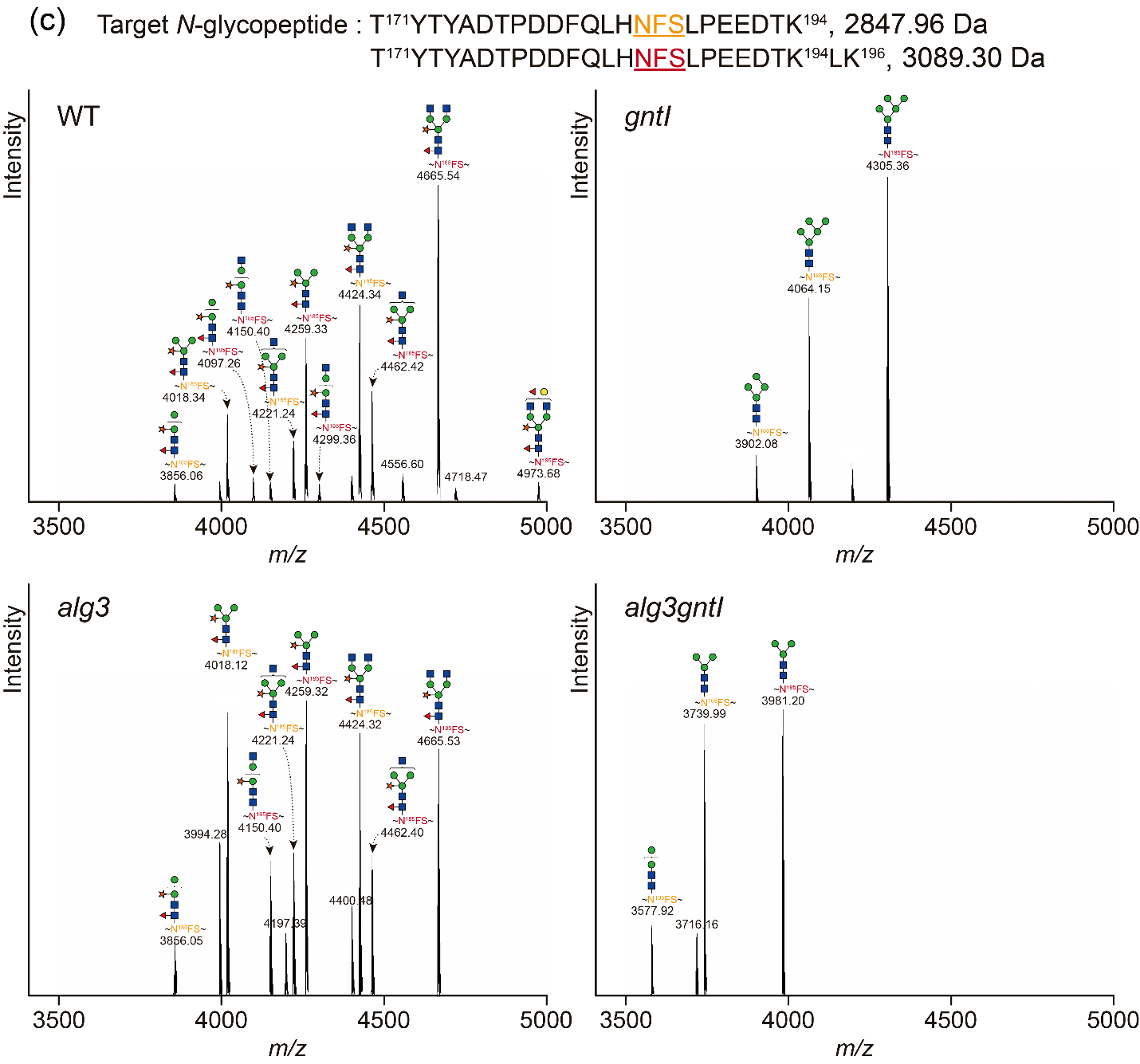
**

**Supplementary Figure S6,** Continued


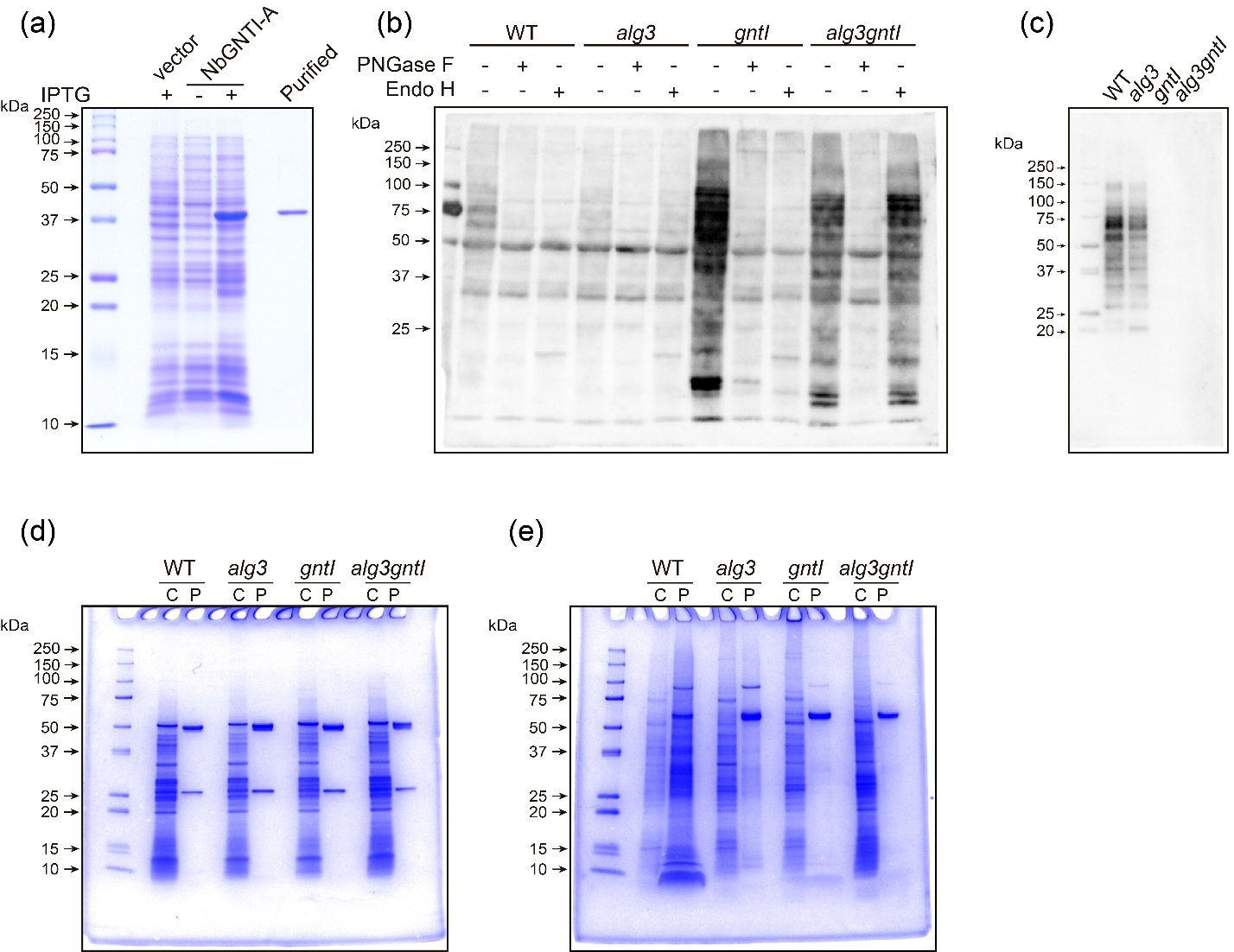


**Supplementary Figure S7** Uncropped images of Figures (a)3a, (b)5b, (c)5c, (d)7a, and (e)8a.

**Supplementary Table 1** **Purification yield of Varlilumab from *N. benthamiana mutants*.**

This table summarizes the yield of purified Varlilumab expressed in different *N. benthamiana* plants. The yields were calculated based on the amount of antibody purified using Protein A spin column from equal amounts of leaf sample.

|  | Fresh leaf amount, g | Crude sample volume, µl | Crude sample protein concentration, µg/µL | Total eluted antibody, µg | Purification yield per g fresh biomass, µg/g | Purification yield per mg total soluble protein, µg/mg |
| --- | --- | --- | --- | --- | --- | --- |
| WT | 0.4  0.4  0.4  0.4 | 1000 | 3.5 | 21 | 52.5 | 6.0 |
| *alg3* |  | 1000 | 6.6 | 68 | 136.0 | 10.3 |
| *gnt1* |  | 1000 | 5.9 | 36 | 90.0 | 6.1 |
| *alg3gnt1* |  | 1000 | 3.9 | 68 | 195.0 | 17.4 |
